# Nrf2 Modulation by Natural Compounds in Aging, Neurodegeneration, and Neuropathic Pain

**DOI:** 10.3390/pharmaceutics18010118

**Published:** 2026-01-16

**Authors:** Jurga Bernatoniene, Dalia M. Kopustinskiene, Roberto Casale, Alessandro Medoro, Sergio Davinelli, Luciano Saso, Kestutis Petrikonis

**Affiliations:** 1 Department of Drug Technology and Social Pharmacy, Faculty of Pharmacy, Medical Academy, Lithuanian University of Health Sciences, Sukileliu Pr. 13, LT-50161 Kaunas, Lithuania; 2 Institute of Pharmaceutical Technologies, Faculty of Pharmacy, Medical Academy, Lithuanian University of Health Sciences, Sukileliu Pr. 13, LT-50161 Kaunas, Lithuania; daliamarija.kopustinskiene@lsmu.lt; 3 OPUSMEDICA Persons, Care & Research-NPO, 29121 Piacenza, Italy; robertocasale@opusmedica.org; 4 Department of Medicine and Health Sciences “V. Tiberio”, University of Molise, 86100 Campobasso, Italy; alessandro.medoro@unimol.it (A.M.); sergio.davinelli@unimol.it (S.D.); 5 Department of Physiology and Pharmacology “Vittorio Erspamer”, La Sapienza University, 00185 Rome, Italy; luciano.saso@uniroma1.it; 6 Department of Neurology, Lithuanian University of Health Sciences, Eivenių Str. 2, LT-50009 Kaunas, Lithuania; kestutis.petrikonis@lsmu.lt

**Keywords:** nuclear factor erythroid 2–related factor 2 (Nrf2), aging, neuroinflammation, oxidative stress, Nrf2 modulators, natural compounds

## Abstract

This review summarizes the role of nuclear factor erythroid 2–related factor 2 (Nrf2) as a common link between aging, neurodegeneration, and neuropathic pain. Aging is characterized by oxidative stress and constant inflammation, which coincides with reduced Nrf2 activity and weaker antioxidant responses, increasing vulnerability to diseases. In neurodegenerative disorders—including Alzheimer’s, Parkinson’s, Huntington’s disease, and amyotrophic lateral sclerosis—evidence indicates that impaired Nrf2 signaling contributes to oxidative damage, neuroinflammation, and mitochondrial dysfunction. Furthermore, in neuropathic pain, similar mechanisms are involved, and Nrf2 could play a role as a potential analgesic target because of its role in regulating cellular defense pathways. We also review natural Nrf2 modulators (e.g., flavonoids, other polyphenols, terpenoids, alkaloids), discussing their benefits alongside common translational limitations such as poor solubility, low oral bioavailability, rapid metabolism, and potential safety issues, including possible pro-oxidant effects and chemoresistance. We also outline future directions that should prioritize improving delivery systems, addressing *NRF2/KEAP1* gene variations, evaluating combinations with standard therapies, exploring preventive applications, and defining dosing, treatment duration, and long-term safety. Overall, current evidence indicates that Nrf2 modulation is a practical, cross-cutting approach relevant to healthy aging and disease management.

## 1. Introduction

Aging [1,2], neurodegeneration [3], and neuropathic pain [4] represent a major current research focus in international scientific literature. They are interconnected by a common pathological mechanism characterized by a self-perpetuating cycle; oxidative stress leads to inflammation, which in turn causes cellular damage, ultimately resulting in further oxidative stress. This creates a detrimental feedback loop that accelerates disease progression and symptom severity.

Nuclear factor erythroid 2-related factor 2 (Nrf2) is a transcription factor responsible for the activation of the cellular principal defense mechanisms against this circle, widely linked to numerous chronic diseases [3,5]. Nrf2 regulates over 2000 genes important for redox balance, metabolic homeostasis, and detoxification of reactive oxygen species and xenobiotics, including key endogenous antioxidants like glutathione and thioredoxin [6,7,8,9,10]. Due to its effects on modulating cytoprotective pathways, Nrf2 could be a therapeutic target for conditions characterized by persistent oxidative damage and inflammation, such as aging, neurodegenerative disorders, and neuropathic pain [3,4,11,12,13,14]. This review examines the multifaceted involvement of Nrf2 in these pathological conditions and explores the potential of natural compounds to modulate its activity [3,4,15].

Nrf2 functions as a regulator of cellular defense, encompassing xenobiotic detoxification and modulation of immunomodulatory networks [6,16]. Its interplay with inflammatory mediators, such as the nuclear factor kappa-light-chain-enhancer of activated B cells (NF-κB) pathway, positions it as a target for mitigating inflammation [17,18]. Maintaining a robust Nrf2 response is crucial for cellular resilience, particularly in neural tissues susceptible to oxidative damage, hallmarks of neurodegenerative conditions and chronic pain syndromes [3,4].

Kelch-like ECH-associated protein 1 (Keap1) is an adaptor subunit of Cullin 3-based E3 ubiquitin ligase. The Keap1-Nrf2 pathway serves as a primary defense mechanism against oxidative and electrophilic stress [19,20]. Under cellular stress, sensor cysteines within Keap1 undergo conformational changes, inhibiting Nrf2 ubiquitination and allowing Nrf2 to translocate to the nucleus, where it initiates transcription of antioxidant and cytoprotective genes [19,20,21,22]. This complex regulatory network highlights the role of Nrf2 in cellular adaptation and survival, influencing diverse physiological processes [6]. Dysregulation of Nrf2 activity significantly compromises resilience, contributing to numerous chronic diseases. Age-related decline in Nrf2 activity specifically promotes inflammaging—chronic inflammation characterized by elevated pro-inflammatory cytokines that exacerbate tissue damage, impair repair processes, and increase susceptibility to neurodegenerative pathologies—emphasizing the therapeutic potential of Nrf2 activators in promoting healthy aging [3,5,12,13,23].

Literature for this narrative review was collected through targeted searches in PubMed/MEDLINE, Scopus, and ScienceDirect, supplemented by Google Scholar for backward and forward citation tracking, covering data to 15 November 2025 (English-language only). Search terms combined Nrf2-pathway keywords (Nrf2/NRF2, NFE2L2, KEAP1, ARE, HO-1, NQO1) with domain terms for aging/senescence/inflammaging, neurodegeneration (Alzheimer’s, Parkinson’s, Huntington’s disease, ALS), and neuropathic pain (allodynia, hyperalgesia, nerve injury, diabetic neuropathy), alongside intervention terms for natural compounds and major chemical classes (phytochemicals, flavonoids, polyphenols, terpenoids, alkaloids) and key compounds discussed (curcumin, resveratrol, sulforaphane, quercetin, EGCG, berberine, ginsenosides), with additional queries for translational and formulation aspects (bioavailability, pharmacokinetics, solubility, nanoparticles, liposomes, 3D printing). Records were screened by title and abstract and assessed in full text when required to confirm relevance to Nrf2 modulation (direct pathway measures or established downstream targets) and outcomes pertinent to aging biology, neurodegenerative conditions, or neuropathic pain models or clinical features; reference lists of key papers and relevant reviews were also manually screened to identify additional sources. A total of 240 sources were included in the final synthesis.

## 2. Molecular Mechanisms of the Nrf2 Signaling Pathway

Nrf2 functions as a key regulator of cellular defense mechanisms, extending to antioxidant responses, xenobiotic detoxification, and immunomodulatory networks [6,22,24,25].

### 2.1. Nrf2 Structure and Basal Regulation

Nrf2 is a cap’n’collar basic leucine zipper transcription factor encoded by the *NRF2* gene. Its functional integrity relies on conserved Neh1 to Neh7 regions, critical for DNA binding, interaction with Keap1, and transcriptional activation [19,22,26]. Under basal, non-stressed conditions, Nrf2 levels are maintained very low through rapid and continuous proteasomal degradation. This is primarily mediated by the Keap1– Cullin 3 (Cul3)– RING box protein 1 (RBX1) E3 ubiquitin ligase complex. Keap1 acts as a substrate adaptor, binding to Nrf2 in the cytoplasm and facilitating its ubiquitination. Ubiquitinated Nrf2 is then targeted for degradation, ensuring tight control over its activity in the absence of stress [27,28,29]. This negative regulation by Keap1 prevents excessive Nrf2 activation, which can have deleterious effects, such as contributing to chemoresistance in cancer [26,30,31]. Conversely, moderate and sustained activation of Nrf2, particularly through modulating its interaction with Keap1, offers cytoprotection against various stressors [12,30,32]. This homeostatic mechanism ensures rapid induction of protective genes when required, while preventing the energetic burden and potential dysregulation associated with constitutive activation [6,33].

### 2.2. Keap1-Dependent Regulation and Oxidative Stress Sensing

Keap1 functions as a redox sensor, constantly monitoring the intracellular redox state via reactive cysteine residues (e.g., Cys151, Cys273, and Cys288), which are highly sensitive to electrophilic and oxidative modifications [22,25,34]. Upon exposure to oxidative stress, electrophiles, or certain natural compounds, these critical cysteine residues in Keap1 undergo conformational changes. This disrupts the Keap1–Nrf2 interaction, inhibiting ubiquitination of Nrf2 and preventing its proteasomal degradation. Consequently, newly synthesized Nrf2 translocates to the nucleus, where it can initiate transcription of cytoprotective genes [27,35]. This mechanism ensures that the cell can rapidly upregulate its antioxidant and detoxification systems in response to environmental insults, restoring cellular redox balance and mitigating cellular damage [20,36]. This precise and swift dissociation of Nrf2 from Keap1 underscores the adaptability of this pathway as a primary defense against a spectrum of endogenous and exogenous stressors [19,22,34]. The resultant activation of Nrf2 orchestrates a comprehensive cytoprotective response, including not only direct antioxidant enzyme upregulation but also the modulation of critical inflammatory and immune pathways [17,18,24].

### 2.3. Nuclear Translocation and ARE-Mediated Transcription of Nrf2

**Figure 1:**
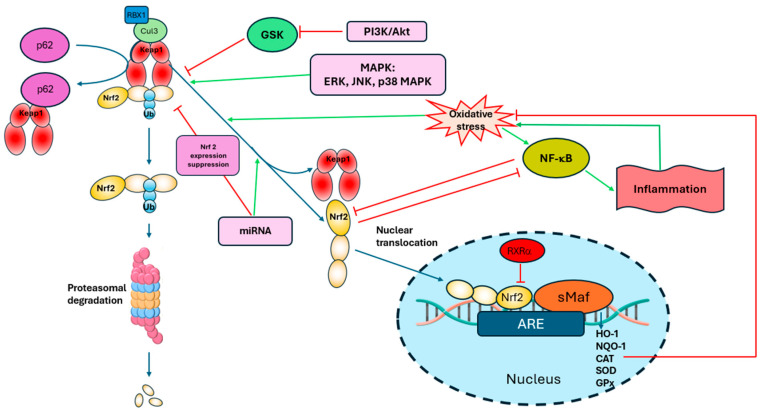
Main Nrf2 regulation pathways. Keap1—Kelch-like ECH-associated protein 1; Cul 3—Cullin3; RBX1—RING-box protein 1; Ub—ubiquitin; PI3K/Akt—phosphoinositide 3-kinase/protein kinase B; ARE—antioxidant response element; sMaf—small Maf proteins; RXRα—retinoid X receptor alpha; NQO-1—NAD(P)H quinone oxidoreductase 1; HO-1—heme-oxygenase-1; CAT—catalase; SOD—superoxide dismutase; GPx—glutathione peroxidase; MAPK—mitogen-activated protein kinase; ERK—extracellular signal-regulated kinase; JNK—c-Jun N-terminal kinase; p38 MAPK—p38 mitogen-activated protein kinase; NF-κB—nuclear factor kappa-light-chain-enhancer of activated B cells.

Once inside the nucleus, Nrf2 does not act alone; it heterodimerizes with small Maf proteins, forming a functional transcription complex [9,37]. This Nrf2-sMaf heterodimer then specifically binds to cis-acting enhancer sequences known as Keap1-Nrf2-Antioxidant Response Element (ARE). These AREs are typically located in the promoter regions of Nrf2-responsive genes. The binding of Nrf2 to AREs drives the transcription of a vast array of cytoprotective genes, including those encoding phase II detoxification enzymes and antioxidant proteins (Figure 1) [8,9,37,38,39,40]. Key examples of these target genes include heme oxygenase-1, NADH quinone oxidoreductase 1, glutamate-cysteine ligase catalytic subunit, glutathione S-transferases, and thioredoxin reductase [9].

This orchestrated gene expression is fundamental to cellular resilience and has implications for various pathological conditions, including neurodegeneration, aging, and neuropathic pain. For instance, Nrf2 activation mitigates UV-induced damage in keratinocytes and human dermal fibroblasts, suggesting its potential in photoprotection and anti-aging strategies [41,42,43]. Moreover, Nrf2 upregulation demonstrably improves wound healing outcomes, especially in conditions characterized by elevated oxidative stress, like diabetes, via activation of its downstream antioxidant genes and promotion of extracellular matrix generation [44,45,46,47]. Furthermore, Nrf2 activation could protect human dermal fibroblasts and endothelial cells from oxidative stress by enhancing antioxidant enzyme activities and preserving cellular viability [18,48].

### 2.4. Alternative Regulatory Mechanisms of Nrf2

While the Keap1-Nrf2 interaction is the primary regulatory axis, Nrf2 activity is also modulated by several Keap1-independent pathways, adding layers of complexity to its control (Figure 1) [6,7]. One such pathway involves Glycogen Synthase Kinase-3 beta (GSK-3β). GSK-3β can phosphorylate Nrf2 at specific serine residues, promoting its degradation independently of Keap1. Inhibition of GSK-3β, therefore, can lead to Nrf2 stabilization and activation (Figure 1) [9].

Another significant regulator is p62/SQSTM1 (sequestosome 1), an autophagy adaptor protein. Under conditions of autophagy impairment or certain cellular stresses, p62 can accumulate and competitively bind to Keap1. This competitive binding sequesters Keap1, preventing it from interacting with Nrf2 and leading to Nrf2 stabilization and prolongation of life. This mechanism links autophagy and Nrf2 signaling [9].

Furthermore, Nrf2 expression and activity are subject to sophisticated epigenetic regulation [7]. MicroRNAs play a critical role in post-transcriptional gene regulation; specific miRNAs (e.g., miR-144 and miR-28) suppress Nrf2 expression, while others indirectly enhance its activity. Histone modifications, such as acetylation and methylation, also influence the accessibility of the *NRF2* gene and its target genes, impacting transcriptional outputof Nrf2 [7,49]. This intricate interplay of Keap1-dependent and independent mechanisms, coupled with epigenetic modulation, ensures a finely tuned cellular response to diverse physiological and pathological stimuli. Recent research also highlights the intricate connections between Nrf2 and ferroptosis, an iron-dependent form of regulated cell death, suggesting a pivotal role of Nrf2 in regulating cellular redox states that govern ferroptosis susceptibility [6,50].

### 2.5. Crosstalk of Nrf2 with Other Signaling Pathways

Nrf2 does not function in isolation; its activity is intricately interconnected with other fundamental cellular signaling pathways, allowing for a comprehensive and integrated cellular response to stress (Figure 1) [6,51]. Significant crosstalk exists between Nrf2 and the NF-κB pathway. While Nrf2 primarily mediates antioxidant and anti-inflammatory responses, NF-κB is a central mediator of pro-inflammatory gene expression. An inverse relationship often exists between their activities; Nrf2 activation can suppress NF-κB activity, thus mitigating inflammation, and vice versa [17,18,52].

The phosphoinositide 3-kinase (PI3K)/AKT pathway is another key regulator. PI3K/AKT activation can lead to Nrf2 activation through phosphorylation events that promote its stability or nuclear translocation, or by modulating upstream regulators like GSK-3β [23].

Similarly, the mitogen-activated protein kinase (MAPK) pathways, including Extracellular Signal-Regulated Kinase (ERK), c-Jun N-terminal kinase (JNK), and p38, can also influence Nrf2 activity. Depending on the context and stimulus, MAPK signaling can activate Nrf2 by promoting its release from Keap1 or its nuclear translocation, or, in some cases, regulate its degradation [23]. This complex interplay ensures a finely tuned cellular response that integrates various stress signals. Furthermore, the interplay between Nrf2 and the circadian clock has been elucidated, revealing that Nrf2 plays a crucial role in the circadian control of mitochondrial reactive oxygen species homeostasis, involving direct transcriptional regulation and sirtuin-dependent posttranslational modifications [6,51]. Redox homeostasis, intricately linked to Nrf2 activity, is a prerequisite for human health, where physiological levels of reactive oxygen species function as vital second messengers in modulating redox signaling [6,51].

### 2.6. Pleiotropic Functions of Nrf2 Beyond Antioxidant Defense

Originally recognized for its pivotal role in antioxidant defense, research has increasingly revealed the pleiotropic functions of Nrf2, demonstrating its influence over a broad spectrum of cellular processes [6]. Nrf2 directly or indirectly regulates over 2000 genes, extending its impact beyond merely counteracting oxidative stress [6,7]. In addition to its established role in activating antioxidant and phase II detoxification enzymes, Nrf2 significantly contributes to anti-inflammatory processes [17,18,24].

Furthermore, Nrf2 plays a crucial role in metabolic pathways, impacting lipid metabolism, glucose homeostasis, and energy production [6,8,10]. It influences mitochondrial function, promoting mitochondrial biogenesis, improving respiratory chain efficiency, and protecting against mitochondrial oxidative damage [6]. Nrf2 also impacts cell survival by upregulating genes involved in cell viability and by modulating processes like autophagy, contributing to cellular resilience and waste removal [6,51]. This multifaceted control positions Nrf2 as a central coordinator of cellular adaptive responses, critical for maintaining overall cellular health and combating various pathophysiological conditions. Its broader implications extend to regulating the ‘redox code’ within cells, which involves dynamic changes in redox states communicating cellular signals and influencing stress responses, thereby maintaining cellular integrity [6,52]. This underscores critical involvement of Nrf2 in modulating the cellular redox environment, which is frequently dysregulated in various pathological conditions such as cancer and chronic inflammatory diseases [6,49]. Given the critical role of reactive oxygen species in cellular signaling and the detrimental effects of their uncontrolled generation, the capacity of Nrf2 to restore redox balance is fundamental for mitigating cellular damage and preventing disease progression [6]. This pathway is also critical in defending against xenobiotics and endogenous toxins, as evidenced by its protective role in organs such as the liver and in conditions like periodontal disease, through mechanisms involving detoxification and inhibition of harmful cellular processes [6,9].

## 3. The Role of Nrf2 in Aging

Aging is characterized by a decline in physiological function and increased disease susceptibility [53]. Aging itself is not a neurodegenerative disease, nevertheless aging and neurodegeneration are intimately related but distinct as normal aging involves gradual, relatively mild cognitive and neural loss and synaptic changes while neurodegenerative diseases (Alzheimer’s, Parkinson’s, ALS, etc.) involve pathological, accelerated loss of specific neuron population with the accumulation of toxic proteins (amyloid, tau, alpha-synuclein, etc). However, there’s growing recognition that aging is the primary risk factor for neurodegeneration and that there are some molecular mechanisms that overlap significantly, such as oxidative stress, mitochondrial dysfunction, inflammation, and a declining Nrf2 activity.

**Figure 2:**
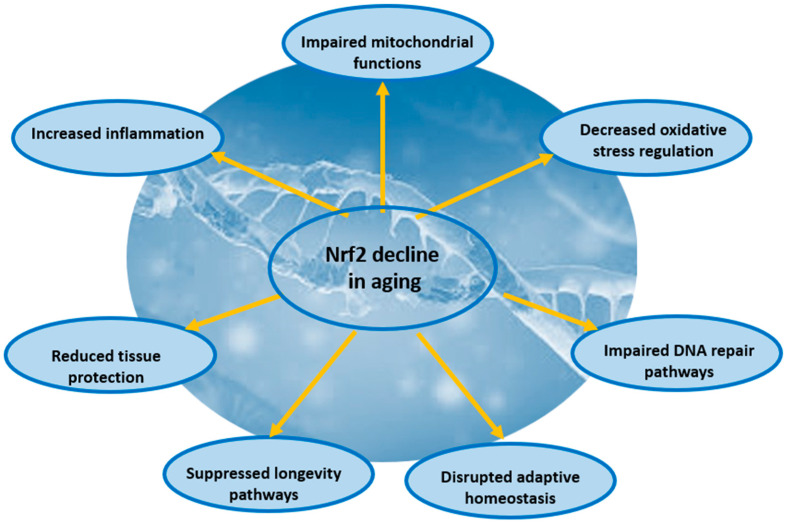
The impact of aging on Nrf2-mediated cytoprotective capacity.

This process involves an accumulation of cellular damage from an imbalance between pro-oxidant and antioxidant activities, which contributes to a chronic, inflammatory state called “inflammaging” [8,54]. Oxidative stress, marked by excess reactive oxygen species, disrupts redox signals and damages cellular components like DNA, proteins, and lipids, contributing to cellular dysfunction and aging [54,55]. The interplay between oxidative stress and inflammation is evident in inflammaging, where persistent inflammation, characterized by elevated pro-inflammatory cytokines, exacerbates tissue damage and impairs cellular repair [12]. The Keap1-Nrf2 system is a crucial defense mechanism that responds to redox perturbations, helping to maintain cellular homeostasis (Figure 2) [22,56].

The Nrf2 pathway is essential for maintaining cellular health and combating oxidative insults [6]. However, its efficiency tends to decline with age due to factors such as increased repressor activity, impaired Nrf2-mediated gene expression, and epigenetic changes [12,15,54].

Diminished Nrf2 function in aging leads to reduced antioxidant defenses, where impaired Nrf2 activity compromises the capacity of cells to upregulate genes that combat oxidative damage, leading to increased oxidative stress [54]. Moreover, impaired Nrf2 activity contributes to stronger inflammation, promoting a chronic inflammatory environment that exacerbates age-related pathologies [12]. Furthermore, Nrf2 contributes to maintaining proteostasis by facilitating the clearance of toxic protein aggregates [12,57]. Its decline with age can impair this process, contributing to neurodegenerative diseases [54,58]. Also, unmitigated oxidative stress can lead to increased DNA damage, affecting telomere shortening and accelerating premature senescence and genomic instability [8,55]. The overall consequence is increased vulnerability to age-related diseases, including neurodegenerative disorders [53].

Given the role of Nrf2 in maintaining cellular homeostasis, its pharmacological modulation represents a strategy for healthy aging [12,53]. Natural compounds are increasingly recognized as Nrf2 activators that can counteract the hallmarks of aging [41,59,60]. By upregulating Nrf2, these compounds contribute to restoring redox balance, where natural Nrf2 activators enhance the expression of cytoprotective and antioxidant enzymes, combating oxidative stress [12]. Also, by strengthening cellular defense mechanisms, natural Nrf2 modulators could help cells withstand stressors encountered during aging [61].

Research into these natural compounds aims to identify agents that can delay or reverse the aging process [62]. The ability of the Keap1-Nrf2 system to prevent aging underscores the therapeutic potential of leveraging natural compounds to maintain cellular health and extend healthspan [63]. This suggests that Nrf2 pathway interventions could be a strategy for promoting healthy aging and longevity.

## 4. Nrf2 in Neurodegenerative Diseases

Neurodegenerative disorders are progressive conditions that lead to the deterioration and death of brain and spinal cord neurons. This loss of neurons causes a progressive and irreversible decline in movement or mental abilities. Examples for epidemiological and symptoms importance include Alzheimer’s and Parkinson’s disease, as well as less common but severe diseases such as Huntington’s disease and lateral amyotrofic sclerosis (ALS). Despite distinct clinical features, these disorders share common pathological hallmarks such as aberrant protein deposition, oxidative stress, neuroinflammation, and mitochondrial dysfunction [3,11,13]. Nrf2, as a regulator of cellular defense against toxic and oxidative insults, plays a protective role in these conditions [3,64,65].

### 4.1. Nrf2 in Alzheimer’s Disease

Alzheimer’s disease (AD) is characterized by amyloid-beta peptide aggregation, hyperphosphorylated tau protein, and redox homeostasis failure [66]. Oxidative stress is an early feature in AD pathogenesis, directly aggravating Aβ deposition and hyperphosphorylated Tau protein [67]. The brain is vulnerable to oxidative stress due to its high oxygen consumption [67].

A decline in Nrf2 function is observed in AD brains [66,68]. This compromised Nrf2 activity diminishes antioxidant defenses, allowing oxidative damage to accumulate. At the cellular level, Nrf2 activation exerts protection of neurons and glia. In neurons, Nrf2 upregulates genes that maintain redox balance, preserve mitochondrial function, and support synaptic resilience. In astrocytes and microglia, Nrf2 shifts transcriptional programs toward antioxidant and anti-inflammatory states, which can indirectly benefit neighboring neurons. In models with astrocyte-biased Nrf2 activation, reductions in oxidative and inflammatory markers often coincide with improved performance in memory tasks, consistent with a network-level benefit from glial support [69].

Conversely, Nrf2 activation has shown therapeutic potential in AD models by activating cytoprotective and antioxidant genes [66,67,68]. Thus, Nrf2 activation is a promising strategy to counteract oxidative stress and Aβ toxicity in AD.

For Alzheimer’s disease, several Nrf2 activators, including sulforaphane, resveratrol, and curcumin, have been evaluated in preclinical models, with some advancing to clinical trials [67]. However, concrete clinical trial outcomes demonstrating significant efficacy in human AD patients are limited [66]. Challenges include a lack of specific Nrf2 activators and suitable AD models for translation [66]. Chromone-containing multitarget-directed ligands, which activate the Nrf2/ARE pathway, are also under evaluation for AD treatment [70].

### 4.2. Nrf2 in Parkinson’s and Huntington’s Diseases

Parkinson’s disease (PD) involves the progressive loss of dopaminergic neurons in the substantia nigra and the formation of α-synuclein-rich Lewy bodies [71]. PD pathogenesis is multifactorial, involving oxidative stress, neuroinflammation, mitochondrial dysfunction, and iron accumulation [71].

Nrf2 is linked to PD pathogenesis, with its regulation influencing these factors [71]. The Nrf2 pathway is often affected in PD patients [72,73]. Nrf2 activation has shown neuroprotective effects in preclinical models, preventing dopaminergic neuronal death by improving redox homeostasis and mitigating mitochondrial damage [71,72,74,75]. Therefore, Nrf2 activators are considered potential therapeutic agents to slow dopaminergic neurodegeneration in PD. Preclinical studies highlight the relevance of Nrf2, and some studies assess Nrf2 pathway components in PD patients as potential biomarkers [76]. However, broad clinical success with Nrf2 activators specifically for PD remains largely in preclinical stages [72].

Huntington’s disease (HD) also involves Nrf2 dysregulation. In HD, oxidative stress plays a role in disease progression, with mitochondrial dysfunction, bioenergetic deficits, and chronic inflammation contributing to a toxic oxidative environment [64,77]. Nrf2 and peroxisome proliferator-activated receptor gamma coactivator-1α (PGC-1α) are key components of cellular defense mechanisms against these insults. Nrf2 activation is crucial for countering oxidative stress in HD [64,77]. While preclinical evidence supports Nrf2 activation as a therapeutic strategy [77,78], the progression to successful human clinical trials for direct Nrf2 activators has been slow [15,79]. Some phytoconstituents have been mentioned in ongoing clinical trials for HD, but specific Nrf2-targeting outcomes are often not detailed [80,81]. The primary hurdle for many promising natural compounds lies in their poor pharmacokinetics and bioavailability, limiting their clinical exploration [15,81]. The field generally faces frustration in translating beneficial Nrf2 modulators from experimental settings to viable therapeutics for neurodegenerative diseases [15,81].

### 4.3. Nrf2 in Amyotrophic Lateral Sclerosis

In ALS, oxidative stress and motor neuron degeneration are central to the disease [82]. The Keap1-Nrf2 system, critical for counteracting oxidative stress, is often impaired [82], as demonstrated both in post-mortem patient tissues [83] and in widely used animal models such as the *SOD1-G93A* transgenic mouse [84]. Studies have shown reduced Nrf2 nuclear translocation and decreased expression of downstream antioxidant genes in motor neurons and surrounding glial cells, which compromises the cellular defense against excessive ROS [82]. This impairment contributes directly to elevated oxidative stress, lipid peroxidation, DNA damage, and protein misfolding, all of which exacerbate motor neuron degeneration [82,83,85]. Additionally, disrupted Nrf2 signaling in astrocytes and microglia promotes a shift toward a pro-inflammatory state, further accelerating neurodegeneration through chronic neuroinflammatory responses [85].

Several experimental studies have demonstrated that pharmacological or genetic activation of Nrf2 can exert neuroprotective effects in ALS models. Activation of Nrf2 increases the expression of cytoprotective enzymes, enhances glutathione synthesis, and improves cellular redox balance, thus reducing oxidative damage [86]. Moreover, Nrf2 activation has been associated with improved mitochondrial function, including enhanced mitochondrial biogenesis, stabilization of mitochondrial membrane potential, and improved ATP production, which are crucial for preserving motor-neuron survival in the metabolically demanding environment of the spinal cord [82,83]. In addition to its antioxidant roles, Nrf2 also modulates inflammatory signaling by suppressing pro-inflammatory cytokine production and promoting a neuroprotective glial phenotype [82]. Collectively, these effects could lead to delayed disease onset, reduced motor neuron loss, and prolonged survival in preclinical ALS models. Therefore, targeting the Nrf2 pathway represents a promising therapeutic strategy aimed at restoring redox homeostasis, reducing neuroinflammation, and improving neuronal resilience in ALS, although translating these findings into effective clinical therapies remains challenging [78].

## 5. Nrf2 and Neuropathic Pain

Neuropathic pain is caused by a lesion or disease of the somatosensory nervous system. It is considered chronic when pain lasts for more than three months, significantly impacting the quality of life of patients. Current pharmacotherapy often provides inadequate relief, highlighting an urgent need for novel therapeutic strategies [4,87]. Nrf2 is recognized as a promising target in this context [4].

### 5.1. The Role of Oxidative Stress and Inflammation in the Pathogenesis and Chronification of Neuropathic Pain

Oxidative stress and neuroinflammation are central to the initiation and chronification of neuropathic pain [4,87]. Oxidative stress arises from an imbalance where reactive oxygen species overwhelm antioxidant systems, leading to cellular damage [16]. In neuropathic pain, excessive ROS contributes to its development [87]. This oxidative imbalance damages nerve cells and modulates signaling pathways [16].

Neuroinflammation, characterized by inflammatory cell activation and mediator production in the nervous system, plays a critical role in the transition to chronic pain [88]. Oxidative stress and inflammation perpetuate each other, exacerbating nerve injury and contributing to chronic pain [89,90]. Increased nitric oxide synthase activity and altered blood-spinal cord barrier integrity, along with astrocyte activation, indicate neuroinflammation [91]. Treatments targeting these inflammatory and oxidative processes can attenuate neuropathic pain [91].

### 5.2. Nrf2 as a Novel Analgesic Target

Given the roles of oxidative stress and neuroinflammation in neuropathic pain, Nrf2, with its ability to coordinate cellular defense processes against these pathological hallmarks, has emerged as an analgesic target [3,4,92]. During peripheral neuropathy, Nrf2 translocates to the nucleus, binding to antioxidant response elements and leading to the transcription of antioxidative enzymes [92]. Nrf2 activation could be an innovative approach to pain management [92]. The Nrf2 transcriptional network has been identified as a potential therapeutic target, for example, in trigeminal neuropathic pain, where cerebrospinal fluid accumulates reactive oxygen species that directly activate pain-transducing channels [93].

Preclinical studies provide evidence for Nrf2 activators, including natural compounds, in various neuropathic pain models. These studies demonstrate that pharmacological agents and natural compounds can be strategies for activating Nrf2 [4]. For instance, the synthetic triterpenoid RTA-408, an Nrf2 activator, significantly reversed mechanical allodynia and thermal hyperalgesia in a chronic constriction injury model of neuropathic pain in mice [94]. This highlights the potential of Nrf2-activating agents inspired by natural sources for pain relief. Other research shows that Nrf2 activation ameliorates neuropathy and neuropathic pain in rodent models by mitigating oxidative stress and neuroinflammation [92].

While preclinical data are robust, the clinical application of Nrf2 activators for neuropathic pain is scarce and contradictory. Existing clinical evidence points to potential benefits of Nrf2 modulators in conditions such as diabetic neuropathy and multiple sclerosis, which can involve neuropathic pain components [4]. For instance, dimethyl fumarate, an Nrf2 activator, is approved for multiple sclerosis, a condition often associated with neuropathic pain [95]. However, the field of Nrf2-targeted therapy for neuropathic pain specifically is largely in preclinical development [87]. Studies evaluating Nrf2 involvement in human diseases, including some related to pain and inflammation, are ongoing, but specific clinical trial results demonstrating Nrf2 activators as direct analgesics for neuropathic pain in humans are limited [96]. More research on detailed mechanisms and dedicated human clinical trials is needed to optimize Nrf2 modulation for pain management [4,87].

## 6. Natural Compounds as Nrf2 Modulators

The Nrf2 pathway is a therapeutic target for various diseases, with natural compounds increasingly recognized as modulators of its activity [97,98]. Numerous phytochemicals, diverse in chemical structure, including flavonoids, other polyphenols, terpenoids, and alkaloids, could activate Nrf2 by influencing the Nrf2 signaling cascade through several complementary mechanisms [59,99,100] (Table 1).

### 6.1. Mechanisms of Nrf2 Modulation by Natural Compounds

**Figure 3:**
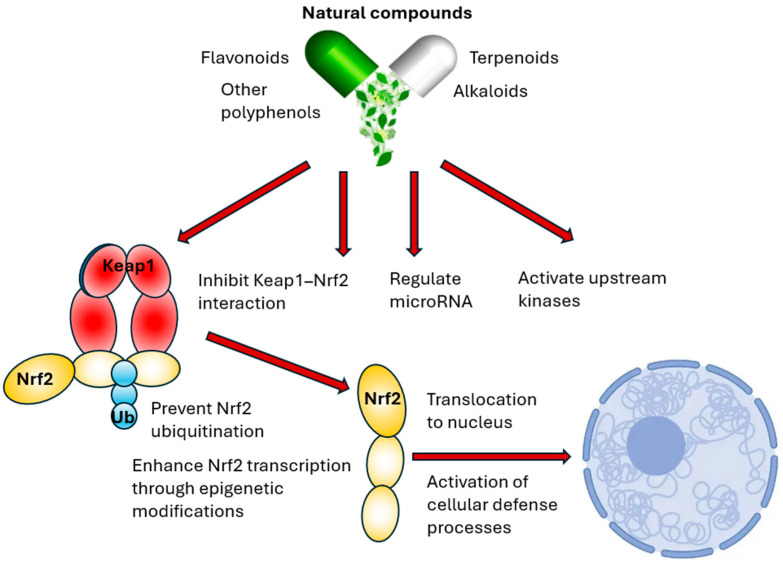
Pathways of Nrf2 modulation by natural compounds.

Natural compounds could activate or modulate the Nrf2 pathway through various mechanisms, such as inhibition of Keap1 interaction, epigenetic regulation, or activation of upstream kinases [73]. Many compounds, particularly electrophiles like sulforaphane, directly modify specific reactive cysteine residues on Keap1. This causes a conformational change in Keap1, preventing it from binding to Nrf2 or facilitating Nrf2 ubiquitination. Nrf2 is then stabilized, accumulates, and translocates to the nucleus, upregulating antioxidant response element-driven genes (Figure 3) [161].

Some natural compounds can influence Nrf2 expression or activity through epigenetic modifications, such as altering DNA methylation patterns or histone modifications in the *NRF2* gene promoter, thereby increasing Nrf2 transcription [162]. They can also modulate microRNA expression, indirectly enhancing Nrf2 levels [7].

Natural compounds could also activate upstream kinases that phosphorylate Nrf2 or its regulatory proteins, leading to Nrf2 stabilization, nuclear translocation, or enhanced transcriptional activity [161]. This phosphorylation can alter the affinity of Nrf2 for Keap1 or enhance its interaction with co-activators, augmenting its transcriptional output [37].

### 6.2. Nrf2 and Flavonoids

Flavonoids are polyphenolic compounds found in fruits, vegetables, and beverages. Many flavonoids are known for their antioxidant, anti-inflammatory, and neuroprotective properties, primarily due to their Nrf2 modulating capabilities [163,164]. Flavones such as apigenin and luteolin modulate Nrf2 and NF-κB pathways, contributing to their anti-inflammatory and antioxidant effects. Other flavonoids, including fisetin and kaempferol, exhibit neuroprotective and anti-inflammatory actions by upregulating Nrf2 and its downstream targets. These compounds are abundant in dietary sources and influence cellular defense mechanisms.

Quercetin, which is abundant in apples, onions, and berries, could activate the Nrf2 pathway, leading to the upregulation of antioxidant enzymes and providing protection against oxidative stress and inflammation [60]. Apigenin, present in parsley, celery, and chamomile, could activate Nrf2, contributing to increased antioxidant and detoxifying enzymes and offering protection against insults like oxidative injury [163,165]. Fisetin, found in strawberries, could demonstrate neuroprotective effects by activating Nrf2, mitigating oxidative stress, inflammation, and cellular senescence [166]. Epigallocatechin gallate, a catechin present in green tea, induces Nrf2 by interacting with Keap1, causing conformational changes that release Nrf2 [134,167,168]. Flavanone naringenin could activate Nrf2, demonstrating antioxidant and anti-inflammatory properties that reduce oxidative stress and mitigate inflammation, contributing to cellular protection [169,170,171,172,173,174].

### 6.3. Nrf2 and Other Polyphenols

Other polyphenolic compounds, including stilbenes, lignans, phenolic acids, and curcuminoids, have received growing attention due to their ability to modulate the Nrf2–Keap1 signaling pathway [166,175,176]. Among stilbenes, resveratrol, a natural polyphenol predominantly found in grapes, red wine, peanuts, and berries, is one of the most extensively studied Nrf2 activators. Resveratrol has been shown to promote Nrf2 nuclear translocation and enhance the expression of downstream antioxidant enzymes such as heme oxygenase-1 (HO-1), NAD(P)H quinone oxidoreductase-1 (NQO1), and glutamate-cysteine ligase (GCL), thereby strengthening the cellular defense against oxidative stress [177,178,179]. In vitro and in vivo studies demonstrate that resveratrol reduces ROS accumulation, suppresses pro-inflammatory mediators (e.g., NF-κB and TNF-α), and improves mitochondrial function through Nrf2-dependent and Nrf2-independent mechanisms, highlighting its role in cellular resilience against oxidative and inflammatory damage.

Another important group of polyphenols is the curcuminoids, with curcumin—the principal bioactive compound of turmeric (*Curcuma longa*)—being the most prominent representative. Curcumin activates Nrf2 primarily by covalently modifying specific cysteine residues on Keap1, the cytosolic repressor of Nrf2, thereby disrupting Nrf2 degradation and facilitating its nuclear accumulation [180,181]. This results in the transcriptional upregulation of numerous cytoprotective genes, including HO-1, superoxide dismutase (SOD), catalase, and glutathione-related enzymes. Beyond its antioxidant action, curcumin exhibits strong anti-inflammatory properties by inhibiting NF-κB signaling and reducing pro-inflammatory cytokine production, making it a promising compound in the prevention and treatment of chronic inflammatory and neurodegenerative diseases [180,181,182].

Phenolic acids, such as ferulic acid, also contribute significantly to Nrf2 activation. Ferulic acid is widely present in cereals (rice, wheat, oats), vegetables, and coffee. Studies have shown that ferulic acid activates the Nrf2/HO-1 signaling pathway, resulting in reduced oxidative stress and inflammation in various cellular and animal models [166,183]. Through this mechanism, ferulic acid enhances cellular antioxidant capacity, inhibits lipid peroxidation, and protects against oxidative damage in models of neurodegeneration, cardiovascular disease, and metabolic disorders. Additionally, its ability to modulate inflammatory signaling further supports its therapeutic potential.

Lignans, a class of polyphenols abundant in flaxseeds, sesame seeds, whole grains, and legumes, have also been identified as Nrf2 activators. Dietary lignans, such as secoisolariciresinol diglucoside (SDG) from flaxseed, are metabolized by intestinal microbiota into enterolignans (enterodiol and enterolactone), which exert antioxidant and anti-inflammatory effects. Evidence suggests that these lignan metabolites promote Nrf2 activation, leading to increased expression of detoxifying and antioxidant enzymes and reduced oxidative damage in various experimental models [166,184]. Their ability to regulate redox homeostasis also contributes to their potential protective effects against cardiovascular disease, cancer, and neurodegenerative conditions.

### 6.4. Nrf2 and Terpenoids

Terpenoids are a class of organic compounds found in essential oils and plants, often exhibiting antioxidant and anti-inflammatory properties linked to Nrf2 upregulation [185]. Abundant in cruciferous vegetables like broccoli, sulforaphane is a potent Nrf2 activator. It modifies specific reactive cysteine residues in Keap1, disrupting the Keap1-Nrf2 interaction, leading to Nrf2 release and sustained upregulation of Nrf2-dependent cytoprotective genes [186,187,188,189,190,191]. A component of oregano and thyme essential oils, carvacrol could activate Nrf2, contributing to its anti-inflammatory and antioxidant activities [192,193,194]. Limonene from citrus fruits could promote Nrf2 activation, enhancing detoxification and antioxidant defense [195,196]. Ginkgolides from *Ginkgo biloba* could activate Nrf2, enhance antioxidant enzyme expression, and induce neuroprotection [197,198,199].

### 6.5. Nrf2 and Alkaloids

Alkaloids, natural compounds with basic nitrogen atoms, often exhibit pharmacological activities through NRF2 activation [200]. An isoquinoline alkaloid, berberine, could activate the Nrf2 pathway, leading to upregulation of cytoprotective genes and providing antioxidant and anti-inflammatory effects [201,202]. Piperine from black pepper could modulate Nrf2 and NF-κB pathways, demonstrating anti-cancer, antioxidant, and anti-inflammatory activities [203]. Geniposide from *Gardenia jasminoides* could activate Nrf2 signaling, mitigating hyperglycemia-induced oxidative stress and inflammation [204,205,206,207,208,209]. Tetrandrine from *Stephania tetrandra* could induce Nrf2 expression, ameliorating oxidative stress and inflammation [166,210].

## 7. Clinical Perspectives and Challenges

Translating Nrf2 modulation into clinical applications faces challenges in pharmacokinetics, bioavailability, and long-term safety [14,15]. The clinical landscape for Nrf2 activators is evolving. Dimethyl fumarate is the most successful Nrf2-targeting therapeutic currently in clinical use, approved for multiple sclerosis and psoriasis [95]. Its efficacy in multiple sclerosis demonstrates Nrf2’s role in mitigating neuroinflammation and oxidative stress in a neurological context [95]. While numerous phytochemicals show Nrf2-activating properties in preclinical settings, evidence of their efficacy and Nrf2 modulation in human clinical trials remains limited (Table 2) [95,211].

Beyond dimethyl fumarate, other electrophilic and non-electrophilic Nrf2 activators are in various stages of clinical development for a range of chronic diseases [14,98]. For example, bardoxolone methyl, another synthetic Nrf2 activator, has been evaluated in clinical trials for chronic kidney disease [14]. Clinical research on small molecule compounds targeting Nrf2 is also progressing for inflammation-related diseases, including some conditions like rheumatoid arthritis, which can involve pain components [219]. Despite this progress, a critical consideration for some Nrf2 activators, including natural compounds like sulforaphane and curcumin, is their mechanism of action, which can involve increasing oxidative stress to activate Nrf2. This raises concerns about potentially counterbalancing benefits, especially in cells already compromised by disease [220]. This dualistic nature complicates therapeutic strategies, as the “timing” of Nrf2 activation can be critical [33].

From a clinical standpoint, the most informative data on ‘how much, how often, and for how long’ Nrf2 should be activated come from approved or late-stage agents such as dimethyl fumarate and omaveloxolone, rather than from nutraceuticals [221,222,223]. Dimethyl fumarate, administered at 240 mg twice daily in relapsing–remitting multiple sclerosis, reliably induces an Nrf2-related transcriptional program, reduces neuroinflammation and oxidative stress, and improves clinical and MRI outcomes, while requiring monitoring for gastrointestinal intolerance, flushing, lymphopenia, and opportunistic infections; this illustrates that clinically relevant Nrf2 activation occurs within a relatively narrow therapeutic and safety window that must be managed with routine laboratory and clinical follow-up [222,223]. Bardoxolone methyl and the related synthetic triterpenoid omaveloxolone, approved for Friedreich’s ataxia at 150 mg once daily, also provide strong systemic Nrf2 activation together with improvements in functional endpoints, but bardoxolone methyl increased cardiovascular events in advanced chronic kidney disease and omaveloxolone requires careful monitoring of hepatic enzymes and lipid profile, underscoring that more intense or sustained Nrf2 activation does not necessarily translate into better clinical outcomes and may unmask organ-specific adverse effects [221,224]. In contrast, for dietary and botanical Nrf2 modulators such as sulforaphane, curcumin, resveratrol, or ginsenosides, human studies usually employ fixed oral doses derived from nutritional or small phase I–II trials but show highly variable bioavailability, rapid metabolism, and short half-lives, without a validated plasma or tissue threshold that defines a ‘therapeutic’ Nrf2 response in brain or peripheral nerves [95,191,225,226,227].

A significant obstacle for natural Nrf2 activators is their unfavorable pharmacokinetic profiles. Most natural Nrf2 modulators exhibit poor solubility, extensive pre-systemic metabolism, low oral bioavailability, and rapid elimination [228]. These limitations restrict therapeutic efficacy and necessitate sophisticated formulation strategies. Challenges such as poor membrane permeability, in vitro and in vivo instability, and a short half-life further complicate clinical application [228]. To address pharmacokinetic and bioavailability challenges, innovative drug delivery systems are being developed to enhance the therapeutic benefits of Nrf2 modulators. Nanotechnology offers promising possibilities for improving delivery and efficacy [228]. Nanodrug delivery systems enhance intracellular uptake and target specificity [199]. Various nanoformulations, including solid lipid nanoparticles, nano-emulsions, nano-crystals, nano-polymersomes, liposomes, ethosomes, and phytosomes, are explored for dietary polyphenols [229]. Albumin-based nanocarriers show promise for simultaneous delivery of antioxidant genes and phytochemicals [230]. These advancements aim to overcome poor solubility and low bioavailability [228]. Three-dimensional printing offers personalized drug delivery, combining multiple doses into a single form tailored to patient genomics [231]. Exploration of 3D and 4D printed dosage forms could provide precision in drug release and facilitate personalized therapeutic strategies, particularly for neurodegenerative diseases.

While Nrf2 activation is generally considered cytoprotective, sustained or aberrant Nrf2 activation can have harmful effects [232]. In cancer, Nrf2 activation can confer resistance to chemotherapy or radiotherapy [211,233]. This is because Nrf2, while protecting healthy cells, can also protect malignant cells from DNA damage, and in some tumors, it is permanently upregulated [233]. Nrf2 can also activate oncogenes unrelated to its antioxidant activity [234]. This “Janus face” of Nrf2 highlights the need for careful consideration when designing Nrf2-targeting therapies [95]. Furthermore, for chronic conditions, some Nrf2 activators increase oxidative stress as part of their activation mechanism, raising concerns about counterbalancing benefits in compromised cells [220]. Therefore, careful evaluation of long-term effects and safety profiles is critical for clinical translation [5]. The context-specific role of Nrf2 in diseases and potential off-target effects remain areas of ongoing research [5,33].

## 8. Future Directions

Understanding the role of Nrf2 in cellular defense and its implications across various pathologies, from aging to neurodegeneration and pain, opens possibilities for future research and clinical translation. Overcoming current challenges and developing new approaches will be essential to use Nrf2 modulation effectively.

Individual genetic properties influence the response to Nrf2 modulators. Polymorphisms in the *NRF2* gene and *KEAP1* gene can alter Nrf2 activity and impact disease susceptibility and therapeutic outcomes [235]. Functional Nrf2 polymorphisms are associated with the risk of various human diseases [235]. Therefore, it could be important to identify genetic biomarkers for the fast detection of genetic polymorphisms in *NRF2* and *KEAP1* that predict individual responses to Nrf2-activating therapies [236]. Furthermore, the genetic information could be used to analyze patient populations, enabling tailored therapeutic approaches for maximum efficacy and minimal adverse effects [237,238]. Moreover, the dosage could be adjusted or Nrf2 activators could be selected based on individual genetic profiles for optimal Nrf2 activation [236]. Understanding these genetic variations can lead to more precise disease intervention strategies [235].

While natural compounds and synthetic activators show promise in Nrf2 modulation, future research could explore synergistic combinations to enhance therapeutic efficacy and overcome limitations. Such combinations could lead to more robust Nrf2 activation, target multiple pathways, or lower effective doses, reducing side effects [211]. Also, the strategies could be developed combining Nrf2 modulation with other neuroprotective or analgesic mechanisms to address the complex multifactorial nature of age-related diseases, neurodegeneration, and chronic pain [4].

Given the role of Nrf2 in maintaining cellular homeostasis and combating oxidative stress and inflammation, it holds potential for integration into preventive medicine and healthy aging strategies [12,53]. Furthermore, the role of diet and lifestyle in endogenously activating Nrf2 for health maintenance and disease prevention could be investigated [63]. Reliable biomarkers could be identified to monitor Nrf2 activity in healthy and aging populations, enabling proactive, timely interventions [8,54]. Maintaining Nrf2 efficiency throughout life could be a strategy to mitigate age-associated decline and improve longevity [12].

## 9. Conclusions

Nrf2 links aging, neurodegeneration, and neuropathic pain by coordinating cellular defenses against oxidative stress and inflammation; age-related declines in Nrf2 activity contribute to inflammaging, reduced antioxidant capacity, and increased vulnerability to disease. Across Alzheimer’s, Parkinson’s, Huntington’s disease, ALS, and neuropathic pain, Nrf2 dysregulation promotes oxidative injury and neuroinflammation, identifying this pathway as a shared and mechanistically grounded therapeutic target whose modulation may alleviate disease progression and symptom burden. However, the successful translation of Nrf2-based strategies will depend on overcoming substantial challenges, including limited bioavailability of natural modulators, context-dependent effects of pathway activation, long-term safety concerns, and interindividual genetic variability. Future advances will require interdisciplinary efforts integrating pharmacology, drug-delivery science, genomics, and clinical trial design, as well as the development of precision and combination approaches to achieve controlled and disease-specific Nrf2 modulation. If these obstacles can be addressed, Nrf2-targeted interventions hold strong potential to evolve from experimental concepts into clinically meaningful tools for prevention and therapy across a broad spectrum of chronic neurological and age-related disorders.

## Figures and Tables

**Table 1:** Representative natural compounds reported to modulate Nrf2 signaling in aging, neurodegeneration, and neuropathic pain models.

Compound	Botanical Source	Experimental Model	Principal Mechanism of Nrf2 Activation
Curcumin	*Curcuma longa* (turmeric rhizome)	In vitro neuronal cultures; rodent models of Alzheimer’s disease and neuropathic pain [101,102,103,104,105,106]; limited phase II clinical trials [107,108]	Disruption of Keap1–Nrf2 interaction via thiol modification; activation of ARE transcription
Resveratrol	*Vitis vinifera* (grapes, red wine)	Extensive in vitro and in vivo data [109,110,111,112,113,114]; small-scale clinical studies in aging and cognitive decline [115,116]	Sirtuin 1 (SIRT1)/AMPK-mediated Nrf2 nuclear translocation; suppression of oxidative and inflammatory pathways
Sulforaphane	*Cruciferous vegetables* (e.g., broccoli)	Strong preclinical evidence (rodents, primates) [117,118,119,120,121]; several ongoing human clinical trials [122,123,124,125]	Covalent modification of Keap1 cysteine residues, robust Nrf2 stabilization
Quercetin	Onions, apples, various fruits	Multiple cell culture and animal studies [126,127,128,129,130,131,132]; limited clinical observations [133]	Inhibition of proteasomal Nrf2 degradation; attenuation of ROS and pro-inflammatory cytokines
Epigallocatechin gallate (EGCG)	*Camellia sinensis* (green tea)	In vitro and in vivo models of neurodegeneration [134,135,136,137,138]; preliminary human intervention data [136]	Enhancement of Nrf2-mediated transcription; modulation of mitochondrial redox balance
Berberine	*Berberis vulgaris* (barberry root)	In vitro neuronal and glial cell models; in vivo neuroinflammation studies; exploratory clinical data in metabolic disorders [139,140,141,142,143,144,145]	Activation of Nrf2 via AMPK and PI3K/Akt signaling; regulation of mitochondrial homeostasis
Apigenin	*Apium graveolens* (celery), chamomile	Cell culture studies; rodent neurodegeneration models; limited translational evidence [146,147,148,149,150]	Induction of Nrf2/HO-1 axis; modulation of MAPK and NF-κB signaling
Luteolin	Parsley, celery, green peppers	Extensive preclinical data in neuroinflammation and neuropathic pain [132,151,152,153,154]; scarce human studies [73]	Stabilization of Nrf2 through inhibition of GSK-3β-mediated degradation; anti-inflammatory effects
Ginsenosides (e.g., Rg1, Rb1)	*Panax ginseng* (ginseng root)	Strong in vitro and in vivo neuroprotective evidence [155,156,157,158,159]; several clinical trials in cognitive function [160]	Activation of Nrf2/ARE pathway; attenuation of oxidative stress and neuronal apoptosis

**Table 2:** Clinical and translational evidence of Nrf2-modulating compounds in neurodegenerative and neuropathic conditions.

Compound	Condition/Patient Group	Clinical Stage	Key Findings	Translational Barriers
Curcumin	Alzheimer’s disease	Phase II, randomized controlled [212]	Mild cognitive improvement; reduced oxidative stress	Poor oral bioavailability; variable response
Resveratrol	Aging; mild cognitive impairment	Phase II, placebo-controlled [177,213]	Enhanced cerebral blood flow; memory improvement	Low systemic exposure; heterogeneous results
Sulforaphane	Schizophrenia; autism	Pilot trials [214,215]	Behavioral improvements; redox normalization	Small sample sizes; limited follow-up
EGCG	Parkinson’s disease	Open-label [134]	Reduced oxidative biomarkers; partial motor benefit	Poor adherence; short duration
Ginsenosides	Mild cognitive impairment	Phase II, multicenter [160]	Improved attention and memory	Restricted populations; uncertain mechanism
Quercetin	Peripheral neuropathy (experimental clinical)	Pilot human study [216]	Trends toward pain reduction; antioxidant effect	Limited clinical validation; dosing inconsistency
Baicalin	Cognitive impairment (traditional medicine use)	Observational/exploratory [217,218]	Neuroprotective indications reported in patients	Lack of controlled clinical trials
Melatonin	Neurodegenerative disorders; sleep disturbances	Multiple clinical trials [27]	Improved sleep and antioxidant markers	Mixed cognitive outcomes; dose variability

## Data Availability

All data is included within the manuscript.

## References

[1] Shilovsky G.A., Ashapkin V.V. (2022). Transcription Factor Nrf2 and Mitochondria—Friends or Foes in the Regulation of Aging Rate. Biochemistry.

[2] Cominelli G., Sulas F., Pinto D., Rinaldi F., Favero G., Rezzani R. (2025). Neuro-Nutritional Approach to Neuropathic Pain Management: A Critical Review. Nutrients.

[3] Mayer C., Riera-Ponsati L., Kauppinen S., Klitgaard H., Erler J.T., Hansen S.N. (2024). Targeting the NRF2 pathway for disease modification in neurodegenerative diseases: Mechanisms and therapeutic implications. Front. Pharmacol..

[4] Petrikonis K., Bernatoniene J., Kopustinskiene D.M., Casale R., Davinelli S., Saso L. (2024). The antinociceptive role of Nrf2 in neuropathic pain: From mechanisms to clinical perspectives. Pharmaceutics.

[5] Cuadrado A., Cazalla E., Bach A., Bathish B., Naidu S.D., DeNicola G.M., Dinkova-Kostova A.T., Fernández-Ginés R., Grochot-Przeczek A., Hayes J.D. (2025). Health position paper and redox perspectives—Bench to bedside transition for pharmacological regulation of NRF2 in noncommunicable diseases. Redox Biol..

[6] He F., Ru X., Wen T. (2020). NRF2, a transcription factor for stress response and beyond. Int. J. Mol. Sci..

[7] McCord J.M., Gao B., Hybertson B.M. (2023). The complex genetic and epigenetic regulation of the Nrf2 pathways: A review. Antioxidants.

[8] Medoro A., Saso L., Scapagnini G., Davinelli S. (2024). NRF2 signaling pathway and telomere length in aging and age-related diseases. Mol. Cell. Biochem..

[9] Tonelli C., Chio I.I.C., Tuveson D.A. (2018). Transcriptional regulation by Nrf2. Antioxid. Redox Signal..

[10] Yagishita Y., Gatbonton-Schwager T.N., McCallum M.L., Kensler T.W. (2020). Current landscape of NRF2 biomarkers in clinical trials. Antioxidants.

[11] Brandes M.S., Gray N.E. (2020). NRF2 as a therapeutic target in neurodegenerative diseases. ASN Neuro.

[12] Buttari B., Tramutola A., Rojo A.I., Chondrogianni N., Saha S., Berry A., Giona L., Miranda J.P., Profumo E., Davinelli S. (2025). Proteostasis decline and redox imbalance in age-related diseases: The therapeutic potential of NRF2. Biomolecules.

[13] Cores Á., Piquero M., Villacampa M., León R., Menéndez J.C. (2020). NRF2 regulation processes as a source of potential drug targets against neurodegenerative diseases. Biomolecules.

[14] Lastra D., Fernández-Ginés R., Manda G., Cuadrado A. (2020). Perspectives on the clinical development of NRF2-targeting drugs. Handbook of Experimental Pharmacology.

[15] Chen W.-T., Dodson M. (2023). The untapped potential of targeting NRF2 in neurodegenerative disease. Front. Aging.

[16] Pang L., Lian X., Liu H., Zhang Y., Li Q., Cai Y., Ma H., Yu X. (2020). Understanding diabetic neuropathy: Focus on oxidative stress. Oxid. Med. Cell. Longev..

[17] Kim J., Surh Y.-J. (2009). The role of Nrf2 in cellular innate immune response to inflammatory injury. Toxicol. Res..

[18] Saha S., Buttari B., Panieri E., Profumo E., Saso L. (2020). An overview of Nrf2 signaling pathway and its role in inflammation. Molecules.

[19] Baird L., Yamamoto M. (2020). The molecular mechanisms regulating the KEAP1-NRF2 pathway. Mol. Cell. Biol..

[20] Deshmukh P., Unni S., Krishnappa G., Padmanabhan B. (2017). The Keap1-Nrf2 pathway: Promising therapeutic target to counteract ROS-mediated damage in cancers and neurodegenerative diseases. Biophys. Rev..

[21] Kopacz A., Kloska D., Forman H.J., Jozkowicz A., Grochot-Przeczek A. (2020). Beyond repression of Nrf2: An update on Keap1. Free Radic. Biol. Med..

[22] Suzuki T., Yamamoto M. (2017). Stress-sensing mechanisms and the physiological roles of the Keap1–Nrf2 system during cellular stress. J. Biol. Chem..

[23] Yu C., Xiao J.-H. (2021). The Keap1-Nrf2 system: A mediator between oxidative stress and aging. Oxid. Med. Cell. Longev..

[24] Pant T., Uche N., Juric M., Zielonka J., Bai X. (2024). Regulation of immunomodulatory networks by Nrf2-activation in immune cells: Redox control and therapeutic potential in inflammatory diseases. Redox Biol..

[25] Li W., Kong A.-N. (2009). Molecular mechanisms of Nrf2-mediated antioxidant response. Mol. Carcinog..

[26] Taguchi K., Yamamoto M. (2020). The KEAP1-NRF2 system as a molecular target of cancer treatment. Cancers.

[27] Davinelli S., Medoro A., Savino R., Scapagnini G. (2024). Sleep and Oxidative Stress: Current Perspectives on the Role of NRF2. Cell. Mol. Neurobiol..

[28] Davinelli S., Medoro A., Siracusano M., Savino R., Saso L., Scapagnini G., Mazzone L. (2025). Oxidative stress response and NRF2 signaling pathway in autism spectrum disorder. Redox Biol..

[29] Ebrahimi R., Mohammadpour A., Medoro A., Davinelli S., Saso L., Miroliaei M. (2025). Exploring the links between polyphenols, Nrf2, and diabetes: A review. Biomed. Pharmacother..

[30] Han J., Yang K., An J., Jiang N., Fu S., Tang X. (2022). The role of NRF2 in bone metabolism—Friend or foe?. Front. Endocrinol..

[31] Park C.K., Lee Y., Kim K.H., Lee Z.H., Joo M., Kim H.-H. (2014). Nrf2 is a novel regulator of bone acquisition. Bone.

[32] Che J., Yang X., Jin Z., Xu C. (2023). Nrf2: A promising therapeutic target in bone-related diseases. Biomed. Pharmacother..

[33] Dodson M., de la Vega M.R., Cholanians A.B., Schmidlin C.J., Chapman E., Zhang D.D. (2019). Modulating NRF2 in disease: Timing is everything. Annu. Rev. Pharmacol. Toxicol..

[34] Yamamoto M., Kensler T.W., Motohashi H. (2018). The KEAP1-NRF2 system: A thiol-based sensor-effector apparatus for maintaining redox homeostasis. Physiol. Rev..

[35] Fiori E., Davinelli S., Ferrera A., Medoro A., Barsali C., Battistoni A., Volterrani M., Volpe M., Saso L., Rubattu S. (2025). The emerging role of Nrf2 in heart failure: From cardioprotection to therapeutic approaches. ESC Heart Fail..

[36] Dayalan Naidu S., Dinkova-Kostova A.T. (2020). KEAP1, a cysteine-based sensor and a drug target for the prevention and treatment of chronic disease. Open Biol..

[37] Vomund S., Schäfer A., Parnham M., Brüne B., Von Knethen A. (2017). Nrf2, the master regulator of anti-oxidative responses. Int. J. Mol. Sci..

[38] Davinelli S., Medoro A., Intrieri M., Saso L., Scapagnini G., Kang J.X. (2022). Targeting NRF2-KEAP1 axis by Omega-3 fatty acids and their derivatives: Emerging opportunities against aging and diseases. Free Radic. Biol. Med..

[39] Nguyen T., Nioi P., Pickett C.B. (2009). The Nrf2-antioxidant response element signaling pathway and its activation by oxidative stress. J. Biol. Chem..

[40] Raghunath A., Sundarraj K., Nagarajan R., Arfuso F., Bian J., Kumar A.P., Sethi G., Perumal E. (2018). Antioxidant response elements: Discovery, classes, regulation and potential applications. Redox Biol..

[41] Boo Y.C. (2020). Natural Nrf2 modulators for skin protection. Antioxidants.

[42] Chaiprasongsuk A., Panich U. (2022). Role of phytochemicals in skin photoprotection via regulation of Nrf2. Front. Pharmacol..

[43] Frantz M.-C., Rozot R., Marrot L. (2023). NRF2 in dermo-cosmetic: From scientific knowledge to skin care products. Biofactors.

[44] Hiebert P., Werner S. (2019). Regulation of wound healing by the NRF2 transcription factor-more than cytoprotection. Int. J. Mol. Sci..

[45] Li M., Yu H., Pan H., Zhou X., Ruan Q., Kong D., Chu Z., Li H., Huang J., Huang X. (2019). Nrf2 suppression delays diabetic wound healing through sustained oxidative stress and inflammation. Front. Pharmacol..

[46] Long M., Rojo de la Vega M., Wen Q., Bharara M., Jiang T., Zhang R., Zhou S., Wong P.K., Wondrak G.T., Zheng H. (2016). An essential role of NRF2 in diabetic wound healing. Diabetes.

[47] Victor P., Sarada D., Ramkumar K.M. (2020). Pharmacological activation of Nrf2 promotes wound healing. Eur. J. Pharmacol..

[48] Liu W., Yan F., Xu Z., Chen Q., Ren J., Wang Q., Chen L., Ying J., Liu Z., Zhao J. (2022). Urolithin A protects human dermal fibroblasts from UVA-induced photoaging through NRF2 activation and mitophagy. J. Photochem. Photobiol. B.

[49] Yang X., Liu Y., Cao J., Wu C., Tang L., Bian W., Chen Y., Yu L., Wu Y., Li S. (2025). Targeting epigenetic and post-translational modifications of NRF2: Key regulatory factors in disease treatment. Cell Death Discov..

[50] Li L., Liu X., Si Z., Wang X. (2025). Epigenetic Mechanisms Governing Nrf2 Expression and Its Role in Ferroptosis. Biomedicines.

[51] Mukherjee R., Rana R., Mehan S., Khan Z., Das Gupta G., Narula A.S., Samant R. (2025). Investigating the Interplay Between the Nrf2/Keap1/HO-1/SIRT-1 Pathway and the p75NTR/PI3K/Akt/MAPK Cascade in Neurological Disorders: Mechanistic Insights and Therapeutic Innovations. Mol. Neurobiol..

[52] Michalak K.P., Michalak A.Z. (2025). Understanding chronic inflammation: Couplings between cytokines, ROS, NO, Ca_i_^2+^, HIF-1α, Nrf2 and autophagy. Front. Immunol..

[53] Francisqueti-Ferron F.V., Ferron A.J.T., Garcia J.L., Silva C.C.V.d.A., Costa M.R., Gregolin C.S., Moreto F., Ferreira A.L.A., Minatel I.O., Correa C.R. (2019). Basic concepts on the role of nuclear factor erythroid-derived 2-like 2 (Nrf2) in age-related diseases. Int. J. Mol. Sci..

[54] Schmidlin C.J., Dodson M.B., Madhavan L., Zhang D.D. (2019). Redox regulation by NRF2 in aging and disease. Free Radic. Biol. Med..

[55] Barnes R.P., Fouquerel E., Opresko P.L. (2019). The impact of oxidative DNA damage and stress on telomere homeostasis. Mech. Ageing Dev..

[56] Cardozo L.F.M.F., Pedruzzi L.M., Stenvinkel P., Stockler-Pinto M.B., Daleprane J.B., Leite M., Mafra D. (2013). Nutritional strategies to modulate inflammation and oxidative stress pathways via activation of the master antioxidant switch Nrf2. Biochimie.

[57] Hatanaka A., Nakada S., Matsumoto G., Satoh K., Aketa I., Watanabe A., Hirakawa T., Tsujita T., Waku T., Kobayashi A. (2023). The transcription factor NRF1 (NFE2L1) activates aggrephagy by inducing p62 and GABARAPL1 after proteasome inhibition to maintain proteostasis. Sci. Rep..

[58] Ngo V., Karunatilleke N.C., Brickenden A., Choy W.-Y., Duennwald M.L. (2022). Oxidative stress-induced misfolding and inclusion formation of Nrf2 and Keap1. Antioxidants.

[59] Zhang D.D., Chapman E. (2020). The role of natural products in revealing NRF2 function. Nat. Prod. Rep..

[60] Zhang L., Xu L.-Y., Tang F., Liu D., Zhao X.-L., Zhang J.-N., Xia J., Wu J.-J., Yang Y., Peng C. (2024). New perspectives on the therapeutic potential of quercetin in non-communicable diseases: Targeting Nrf2 to counteract oxidative stress and inflammation. J. Pharm. Anal..

[61] Bruns D.R., Drake J.C., Biela L.M., Peelor F.F., Miller B.F., Hamilton K.L. (2015). Nrf2 signaling and the slowed aging phenotype: Evidence from long-lived models. Oxid. Med. Cell. Longev..

[62] Zinovkin R.A., Kondratenko N.D., Zinovkina L.A. (2022). Does Nrf2 play a role of a master regulator of mammalian aging?. Biochemistry.

[63] Matsumaru D., Motohashi H. (2021). The KEAP1-NRF2 system in healthy aging and longevity. Antioxidants.

[64] D’Egidio F., Qosja E., Ammannito F., Topi S., d’Angelo M., Cimini A., Castelli V. (2025). Antioxidant and anti-inflammatory defenses in Huntington’s disease: Roles of NRF2 and PGC-1α, and therapeutic strategies. Life.

[65] Johnson D.A., Johnson J.A. (2015). Nrf2—A therapeutic target for the treatment of neurodegenerative diseases. Free Radic. Biol. Med..

[66] Osama A., Zhang J., Yao J., Yao X., Fang J. (2020). Nrf2: A dark horse in Alzheimer’s disease treatment. Ageing Res. Rev..

[67] Qu Z., Sun J., Zhang W., Yu J., Zhuang C. (2020). Transcription factor NRF2 as a promising therapeutic target for Alzheimer’s disease. Free Radic. Biol. Med..

[68] Bitra V.R., Moshapa F., Adiukwu P.C., Rapaka D. (2024). Nrf2-mediated signaling as a therapeutic target in Alzheimer’s disease. Open Neurol. J..

[69] Wang T., Sobue A., Watanabe S., Komine O., Saido T.C., Saito T., Yamanaka K. (2024). Dimethyl fumarate improves cognitive impairment and neuroinflammation in mice with Alzheimer’s disease. J. Neuroinflamm..

[70] Simakov A., Chhor S., Ismaili L., Martin H. (2025). Nrf2 activation and antioxidant properties of chromone-containing MTDLs for Alzheimer’s disease treatment. Molecules.

[71] Yang X.-X., Yang R., Zhang F. (2022). Role of Nrf2 in Parkinson’s disease: Toward new perspectives. Front. Pharmacol..

[72] Parga J.A., Rodriguez-Perez A.I., Garcia-Garrote M., Rodriguez-Pallares J., Labandeira-Garcia J.L. (2021). NRF2 activation and downstream effects: Focus on Parkinson’s disease and brain Angiotensin. Antioxidants.

[73] Gote S., Dubey S., Nargund S.L., Thapa S. (2025). A systematic review of natural products targeting Nrf2-Keap1-ARE pathway and their influence on neurodegenerative disorders. Inflammopharmacology.

[74] Izumi Y. (2013). Dopaminergic neuroprotection via Nrf2-ARE pathway activation: Identification of an activator from green perilla leaves. Yakugaku Zasshi.

[75] Niu Y., Zhang J., Dong M. (2021). Nrf2 as a potential target for Parkinson’s disease therapy. J. Mol. Med..

[76] Petrillo S., Schirinzi T., Di Lazzaro G., D’Amico J., Colona V.L., Bertini E., Pierantozzi M., Mari L., Mercuri N.B., Piemonte F. (2019). Systemic activation of Nrf2 pathway in Parkinson’s disease. Mov. Disord..

[77] Tucci P., Lattanzi R., Severini C., Saso L. (2022). Nrf2 pathway in Huntington’s disease (HD): What is its role?. Int. J. Mol. Sci..

[78] Jiménez-Villegas J., Ferraiuolo L., Mead R.J., Shaw P.J., Cuadrado A., Rojo A.I. (2021). NRF2 as a therapeutic opportunity to impact in the molecular roadmap of ALS. Free Radic. Biol. Med..

[79] Arslanbaeva L., Bisaglia M. (2022). Activation of the Nrf2 pathway as a therapeutic strategy for ALS treatment. Molecules.

[80] Singh P., Mishra G., Molla M., Shumet Yimer Y., Sisay W., Andargie Y., Ewunetie A. (2022). Dietary and nutraceutical-based therapeutic approaches to combat the pathogenesis of Huntington’s disease. J. Funct. Foods.

[81] Vishwas S., Gulati M., Kapoor B., Gupta S., Singh S.K., Awasthi A., Khan A., Goyal A., Bansal A., Baishnab S. (2021). Expanding the arsenal against Huntington’s disease-herbal drugs and their nanoformulations. Curr. Neuropharmacol..

[82] Bono S., Feligioni M., Corbo M. (2021). Impaired antioxidant KEAP1-NRF2 system in amyotrophic lateral sclerosis: NRF2 activation as a potential therapeutic strategy. Mol. Neurodegener..

[83] Sharbafshaaer M., Pepe R., Notariale R., Canale F., Tedeschi G., Tessitore A., Bergamo P., Trojsi F. (2025). Beyond Antioxidants: The Emerging Role of Nrf2 Activation in Amyotrophic Lateral Sclerosis (ALS). Int. J. Mol. Sci..

[84] Wang F., Lu Y., Qi F., Su Q., Wang L., You C., Che F., Yu J. (2014). Effect of the human SOD1-G93A gene on the Nrf2/ARE signaling pathway in NSC-34 cells. Mol. Med. Rep..

[85] Tarot P., Lasbleiz C., Liévens J.C. (2024). NRF2 signaling cascade in amyotrophic lateral sclerosis: Bridging the gap between promise and reality. Neural Regen. Res..

[86] Petri S., Körner S., Kiaei M. (2012). Nrf2/ARE Signaling Pathway: Key Mediator in Oxidative Stress and Potential Therapeutic Target in ALS. Neurol. Res. Int..

[87] Zhou Y.-Q., Mei W., Tian X.-B., Tian Y.-K., Liu D.-Q., Ye D.-W. (2021). The therapeutic potential of Nrf2 inducers in chronic pain: Evidence from preclinical studies. Pharmacol. Ther..

[88] Wen B., Pan Y., Cheng J., Xu L., Xu J. (2023). The role of neuroinflammation in complex Regional Pain Syndrome: A comprehensive review. J. Pain Res..

[89] Sandireddy R., Yerra V.G., Areti A., Komirishetty P., Kumar A. (2014). Neuroinflammation and oxidative stress in diabetic neuropathy: Futuristic strategies based on these targets. Int. J. Endocrinol..

[90] Teixeira-Santos L., Albino-Teixeira A., Pinho D. (2020). Neuroinflammation, oxidative stress and their interplay in neuropathic pain: Focus on specialized pro-resolving mediators and NADPH oxidase inhibitors as potential therapeutic strategies. Pharmacol. Res..

[91] Sharma H.S., Feng L., Chen L., Huang H., Ryan Tian Z., Nozari A., Muresanu D.F., Lafuente J.V., Castellani R.J., Wiklund L. (2023). Cerebrolysin attenuates exacerbation of neuropathic pain, blood-spinal cord barrier breakdown and cord pathology following chronic intoxication of engineered Ag, Cu or Al (50–60 nm) nanoparticles. Neurochem. Res..

[92] Basu P., Averitt D.L., Maier C., Basu A. (2022). The effects of nuclear factor erythroid 2 (NFE2)-related factor 2 (Nrf2) activation in preclinical models of peripheral neuropathic pain. Antioxidants.

[93] Vasavda C., Xu R., Liew J., Kothari R., Dhindsa R.S., Semenza E.R., Paul B.D., Green D.P., Sabbagh M.F., Shin J.Y. (2022). Identification of the NRF2 transcriptional network as a therapeutic target for trigeminal neuropathic pain. Sci. Adv..

[94] Sun J., Li J.-Y., Zhang L.-Q., Li D.-Y., Wu J.-Y., Gao S.-J., Zhou Y.-Q., Mei W. (2021). Nrf2 activation attenuates chronic constriction injury-induced neuropathic pain via induction of PGC-1α-mediated mitochondrial biogenesis in the spinal cord. Res. Sq..

[95] Dinkova-Kostova A.T., Copple I.M. (2023). Advances and challenges in therapeutic targeting of NRF2. Trends Pharmacol. Sci..

[96] Staurengo-Ferrari L., Badaro-Garcia S., Hohmann M.S.N., Manchope M.F., Zaninelli T.H., Casagrande R., Verri W.A. (2018). Contribution of Nrf2 modulation to the mechanism of action of analgesic and anti-inflammatory drugs in pre-clinical and clinical stages. Front. Pharmacol..

[97] Matzinger M., Fischhuber K., Heiss E.H. (2018). Activation of Nrf2 signaling by natural products-can it alleviate diabetes?. Biotechnol. Adv..

[98] Robledinos-Antón N., Fernández-Ginés R., Manda G., Cuadrado A. (2019). Activators and inhibitors of NRF2: A review of their potential for clinical development. Oxid. Med. Cell. Longev..

[99] Wu K.C., McDonald P.R., Liu J., Klaassen C.D. (2014). Screening of natural compounds as activators of the keap1-nrf2 pathway. Planta Med..

[100] Singh S., Nagalakshmi D., Sharma K.K., Ravichandiran V. (2021). Natural antioxidants for neuroinflammatory disorders and possible involvement of Nrf2 pathway: A review. Heliyon.

[101] Elsayed H.R.H., Rabei M.R., Elshaer M.M.A., El Nashar E.M., Alghamdi M.A., Al-Qahtani Z., Nabawy A. (2023). Suppression of neuronal apoptosis and glial activation with modulation of Nrf2/HO-1 and NF-kB signaling by curcumin in streptozotocin-induced diabetic spinal cord central neuropathy. Front. Neuroanat..

[102] Li X., Sung P., Zhang D., Yan L. (2023). Curcumin in vitro Neuroprotective Effects Are Mediated by p62/keap-1/Nrf2 and PI3K/AKT Signaling Pathway and Autophagy Inhibition. Physiol. Res..

[103] Wu F., Lin Y., Xiao L., Chen Q., Lin F., Li R. (2024). Administration with curcumin alleviates spinal cord ischemia-reperfusion injury by regulating anti-oxidative stress and microglia activation-mediated neuroinflammation via Nrf2/NF-κB axis. In Vitro Cell. Dev. Biol. Anim..

[104] Xu Y., Liu Y., Wu Y., Sun J., Lu X., Dai K., Zhang Y., Luo C., Zhang J. (2025). Curcumin Alleviates Microglia-Mediated Neuroinflammation and Neuronal Ferroptosis Following Experimental Subarachnoid Hemorrhage by Modulating the Nrf2/HO-1 Signaling Pathway. Mol. Neurobiol..

[105] Youssef H., Mansour Y.A., El-Leithy E.M.M., Galal M.K., Rashad M.M., Bawish B.M., Tolba E., El-Shammaa M.A. (2025). Neurotoxic and neurobehavioral impacts of silica nanoparticles on brain tissue of albino rats with the potential ameliorative efficacy of liposomal curcumin. J. Mol. Histol..

[106] Zhang X., Cui Y., Song X., Jin X., Sheng X., Xu X., Li T., Chen H., Gao L. (2023). Curcumin alleviates ketamine-induced oxidative stress and apoptosis via Nrf2 signaling pathway in rats’ cerebral cortex and hippocampus. Environ. Toxicol..

[107] Alvarenga L., Salarolli R., Cardozo L., Santos R.S., de Brito J.S., Kemp J.A., Reis D., de Paiva B.R., Stenvinkel P., Lindholm B. (2020). Impact of curcumin supplementation on expression of inflammatory transcription factors in hemodialysis patients: A pilot randomized, double-blind, controlled study. Clin. Nutr..

[108] Karimi A., Naeini F., Niazkar H.R., Tutunchi H., Musazadeh V., Mahmoodpoor A., Asghariazar V., Mobasseri M., Tarighat-Esfanjani A. (2022). Nano-curcumin supplementation in critically ill patients with sepsis: A randomized clinical trial investigating the inflammatory biomarkers, oxidative stress indices, endothelial function, clinical outcomes and nutritional status. Food Funct..

[109] Koszła O., Sołek P., Jóźwiak K. (2025). Co-treatment Strategy Supports Neuroprotection by Intersecting p62-Keap1-NRF2 and Autophagy Signaling Pathways in the Cellular Model of Parkinson’s Disease. Cell. Mol. Neurobiol..

[110] Quincozes-Santos A., Bobermin L.D., Tramontina A.C., Wartchow K.M., Da Silva V.F., Gayger-Dias V., Thomaz N.K., de Moraes A.D.M., Schauren D., Nardin P. (2025). Glioprotective Effects of Resveratrol Against Glutamate-Induced Cellular Dysfunction: The Role of Heme Oxygenase 1 Pathway. Neurotox. Res..

[111] Sovrani V., Bobermin L.D., Sesterheim P., Rezena E., Cioccari M.S., Netto C.A., Gonçalves C.A., Leipnitz G., Quincozes-Santos A. (2023). Glioprotective effects of resveratrol in hypothalamic astrocyte cultures obtained from interferon receptor knockout (IFNα/βR^−/−^) mice. Vitr. Cell. Dev. Biol. Anim..

[112] Thiel G., Rössler O.G. (2017). Resveratrol regulates gene transcription via activation of stimulus-responsive transcription factors. Pharmacol. Res..

[113] Zamanian M.Y., Parra R.M.R., Soltani A., Kujawska M., Mustafa Y.F., Raheem G., Al-Awsi L., Lafta H.A., Taheri N., Heidari M. (2023). Targeting Nrf2 signaling pathway and oxidative stress by resveratrol for Parkinson’s disease: An overview and update on new developments. Mol. Biol. Rep..

[114] Zheng Q., Huang D., Zhao L., Long X., Tu Q., Song L., Wang J., Zheng W., Wen X., Zhang C. (2025). Groundbreaking Insights Into SIRT1/NRF2-Mediated Ferroptosis Inhibition by Resveratrol in Parkinson’s Disease Models. CNS Neurosci. Ther..

[115] Saldanha J.F., Leal V.O., Rizzetto F., Grimmer G.H., Ribeiro-Alves M., Daleprane J.B., Carraro-Eduardo J.C., Mafra D. (2016). Effects of Resveratrol Supplementation in Nrf2 and NF-κB Expressions in Nondialyzed Chronic Kidney Disease Patients: A Randomized, Double-Blind, Placebo-Controlled, Crossover Clinical Trial. J. Ren. Nutr..

[116] Seyyedebrahimi S., Khodabandehloo H., Nasli Esfahani E., Meshkani R. (2018). The effects of resveratrol on markers of oxidative stress in patients with type 2 diabetes: A randomized, double-blind, placebo-controlled clinical trial. Acta Diabetol..

[117] Goodfellow M.J., Borcar A., Proctor J.L., Greco T., Rosenthal R.E., Fiskum G. (2020). Transcriptional activation of antioxidant gene expression by Nrf2 protects against mitochondrial dysfunction and neuronal death associated with acute and chronic neurodegeneration. Exp. Neurol..

[118] Jeong H., Choi H., Park Y.S. (2025). Neuroprotective Potential of Broccoli Sprout Extract in Scopolamine-Induced Memory-Impaired Mice. Foods.

[119] Moretti D., Tambone S., Cerretani M., Fezzardi P., Missineo A., Sherman L.T., Munoz-Sajuan I., Harper S., Dominquez C., Pacifici R. (2021). NRF2 activation by reversible KEAP1 binding induces the antioxidant response in primary neurons and astrocytes of a Huntington’s disease mouse model. Free Radic. Biol. Med..

[120] Tufekci K.U., Ercan I., Isci K.B., Olcum M., Tastan B., Gonul C.P., Genc K., Genc S. (2021). Sulforaphane inhibits NLRP3 inflammasome activation in microglia through Nrf2-mediated miRNA alteration. Immunol. Lett..

[121] Villavicencio-Tejo F., Olesen M.A., Ampuero E., Quintanilla R.A. (2025). Sulforaphane prevents cognitive decline and mitochondrial failure induced by hippocampal expression of caspase-3 cleaved tau. Neurochem. Int..

[122] Beal M.F. (2009). Therapeutic approaches to mitochondrial dysfunction in Parkinson’s disease. Park. Relat. Disord..

[123] Clifford T., Acton J.P., Cocksedge S.P., Davies K.A.B., Bailey S.J. (2021). The effect of dietary phytochemicals on nuclear factor erythroid 2-related factor 2 (Nrf2) activation: A systematic review of human intervention trials. Mol. Biol. Rep..

[124] Fahey J.W., Kensler T.W. (2021). The Challenges of Designing and Implementing Clinical Trials with Broccoli Sprouts… and Turning Evidence into Public Health Action. Front. Nutr..

[125] Kim J. (2021). Pre-Clinical Neuroprotective Evidences and Plausible Mechanisms of Sulforaphane in Alzheimer’s Disease. Int. J. Mol. Sci..

[126] Bahar E., Kim J.Y., Yoon H. (2017). Quercetin Attenuates Manganese-Induced Neuroinflammation by Alleviating Oxidative Stress through Regulation of Apoptosis, iNOS/NF-κB and HO-1/Nrf2 Pathways. Int. J. Mol. Sci..

[127] Bayazid A.B., Lim B.O. (2022). Quercetin Is An Active Agent in Berries against Neurodegenerative Diseases Progression through Modulation of Nrf2/HO1. Nutrients.

[128] Cumaoğlu A., Ağkaya A., Özkul Z. (2019). Effect of the Lipid Peroxidation Product 4-Hydroxynonenal on Neuroinflammation in Microglial Cells: Protective Role of Quercetin and Monochloropivaloylquercetin. Turk. J. Pharm. Sci..

[129] Hussein R.M., Kandeil M.A., Soliman H.M., El-Shahawy A.A.G. (2024). Effect of quercetin-loaded poly (lactic-co-glycolic) acid nanoparticles on lipopolysaccharide-induced memory decline, oxidative stress, amyloidogenesis, neurotransmission, and Nrf2/HO-1 expression. Heliyon.

[130] Jiang Y., Xie G., Alimujiang A., Xie H., Yang W., Yin F., Huang D. (2023). Protective Effects of Querectin against MPP^+^-Induced Dopaminergic Neurons Injury via the Nrf2 Signaling Pathway. Front. Biosci..

[131] Rahmatkar S.N., Rana A.K., Kumar R., Singh D. (2024). *Fagopyrum tataricum* (L.) Gaertn interacts with Gsk-3β/Nrf-2 signalling to protect neurotoxicity in a zebrafish model. J. Ethnopharmacol..

[132] Singh N.K., Varshney N. (2025). Therapeutic Applications of Natural Flavonoids Against Alzheimer’s Disease-like Pathology: Special Focus on PI3K/Akt and Nrf2 Signaling Pathways. Curr. Neurovasc. Res..

[133] Bellavite P. (2023). Neuroprotective Potentials of Flavonoids: Experimental Studies and Mechanisms of Action. Antioxidants.

[134] Amin M.A., Zehravi M., Sweilam S.H., Shatu M.M., Durgawale T.P., Qureshi M.S., Durgapal S., Haque M.A., Vodeti R., Panigrahy U.P. (2025). Neuroprotective potential of epigallocatechin gallate in Neurodegenerative Diseases: Insights into molecular mechanisms and clinical Relevance. Brain Res..

[135] Cheng-Chung Wei J., Huang H.C., Chen W.J., Huang C.N., Peng C.H., Lin C.L. (2016). Epigallocatechin gallate attenuates amyloid β-induced inflammation and neurotoxicity in EOC 13.31 microglia. Eur. J. Pharmacol..

[136] Islam M.R., Rauf A., Akter S., Akter H., Al-Imran M.I.K., Islam S., Nessa M., Shompa C.J., Shuvo M.N.R., Khan I. (2025). Epigallocatechin 3-gallate-induced neuroprotection in neurodegenerative diseases: Molecular mechanisms and clinical insights. Mol. Cell. Biochem..

[137] Kim S.M., Suh H.J., Lee W., Kim B., Han S.H., Jung E.Y., Chang Y.B. (2025). Anti-fatigue and antioxidative effects of amino acid (Leu, Gln, Cys)-EGCG complex via NRF2 and PGC-1α pathways: Insights from cellular, animal, and pilot clinical studies. Nutr. Res. Pract..

[138] Wang M., Zhang Y., Wu Q., Ma S., Wang C., Sang J. (2025). Medicine-food homology bioactives in Parkinson’s disease: Multi-target oxidative-stress modulation and translation to dietary supplements. Front. Nutr..

[139] Chen N., Wang X.C., Fan L.L., Zhu Y.H., Wang Q., Chen Y.B. (2022). Berberine Ameliorates Lipopolysaccharide-Induced Cognitive Impairment Through SIRT1/NRF2/NF-κB Signaling Pathway in C57BL/6J Mice. Rejuvenation Res..

[140] Li X., Chen J., Feng W., Wang C., Chen M., Li Y., Chen J., Liu X., Liu Q., Tian J. (2023). Berberine ameliorates iron levels and ferroptosis in the brain of 3 × Tg-AD mice. Phytomedicine.

[141] Li X., Yu H., Liu R., Miao J., Lv J., Yang S., Zhu Y., Chen Y., Lu K., Huang C. (2025). Activation of the Nrf2 Signaling Pathway by Tetrahydroberberine Suppresses Ferroptosis and Enhances Functional Recovery Following Spinal Cord Injury. Mol. Neurobiol..

[142] Ma H., Xing C., Wei H., Li Y., Wang L., Liu S., Wu Q., Sun C., Ning G. (2024). Berberine attenuates neuronal ferroptosis via the AMPK-NRF2-HO-1-signaling pathway in spinal cord-injured rats. Int. Immunopharmacol..

[143] Nematalla H.A., Elharoun M., Abd-Alhaseeb M.M., Sharafeldin H.A., Elsheikh M.A., Abbas H., Elkelish A., Dossouvi K.M., Mehana A.E., Abdelgawad F.E. (2025). Innovative approach in Parkinson’s targeting via berberine-loaded mucoadhesive surface-modified liposomes: A multi-faceted study. BMC Pharmacol. Toxicol..

[144] Singh A., Kumar V., Langeh U., Kapil L., Kaur S., Rana N., Bhattacharya A., Singh R., Bhatti J.S., Singh C. (2024). In-vitro and in-vivo studies of two-drug cocktail therapy targeting chemobrain via the Nrf2/NF-κB signaling pathway. J. Mol. Histol..

[145] Zhang R.L., Lei B.X., Wu G.Y., Wang Y.Y., Huang Q.H. (2023). Protective effects of berberine against β-amyloid-induced neurotoxicity in HT22 cells via the Nrf2/HO-1 pathway. Bioorg. Chem..

[146] Choi E.M., Park S.Y., Suh K.S., Chon S. (2023). Apigenin attenuates tetrabromobisphenol A-induced cytotoxicity in neuronal SK-N-MC cells. J. Environ. Sci. Health A.

[147] Haridevamuthu B., Ranjan Nayak S.P.R., Murugan R., Pachaiappan R., Ayub R., Aljawdah H.M., Arokiyaraj S., Guru A., Arockiaraj J. (2024). Prophylactic effects of apigenin against hyperglycemia-associated amnesia via activation of the Nrf2/ARE pathway in zebrafish. Eur. J. Pharmacol..

[148] Huang Y.B., Tian L.L., Zhu Z.W., Zhou K.G., Lai X., Peng Y.Z., Wu Z., Tong W.F., Wang H., Wang X.J. (2025). Apigenin enhances Nrf2-induced chaperone-mediated autophagy and mitigates α-synuclein pathology: Implications for Parkinson’s disease therapy. Phytomedicine.

[149] Liang H., Zhou X., Zhang J., Xu W., Liu Y., Wang X., Hu Y., Xu R., Li X. (2024). The therapeutic potential of Apigenin in amyotrophic lateral sclerosis through ALDH1A2/Nrf2/ARE signaling. Mol. Med..

[150] Patel M., Singh S. (2022). Apigenin Attenuates Functional and Structural Alterations via Targeting NF-kB/Nrf2 Signaling Pathway in LPS-Induced Parkinsonism in Experimental Rats: Apigenin Attenuates LPS-Induced Parkinsonism in Experimental Rats. Neurotox. Res..

[151] Albrakati A. (2024). The potential neuroprotective of luteolin against acetamiprid-induced neurotoxicity in the rat cerebral cortex. Front. Vet. Sci..

[152] Kim H., Chung S.H., Kim S., Ko S., Jung J.C., Lee H.J., Lee H. (2025). *Chrysanthemum morifolium* extract protects HT22 cells from oxidative stress and enhances sleep via GABAergic modulation: Contribution of luteolin-7-glucoside. J. Sci. Food Agric..

[153] Mahto K., Kuwar O.K., Maloo A., Kalia N. (2025). Therapeutic potential of luteolin in neurodegenerative disorders: Targeting Nrf2, NFĸB, MAPK, and JAK-STAT pathways to combat neuroinflammation and apoptosis. Inflammopharmacology.

[154] Zhang Z.H., Liu J.Q., Hu C.D., Zhao X.T., Qin F.Y., Zhuang Z., Zhang X.S. (2021). Luteolin Confers Cerebroprotection after Subarachnoid Hemorrhage by Suppression of NLPR3 Inflammasome Activation through Nrf2-Dependent Pathway. Oxid. Med. Cell. Longev..

[155] Fu Y., Zhang H., Zhu X., Liang H., Fan L., Su Y., Li W., Li W. (2025). Chronic lipopolysaccharide exposure promotes cognitive impairments by activating TRPC6-AIM2 inflammasome signaling and the regulation of ginsenoside Rg1 in Trpc6^−/−^ mice. Behav. Brain Funct..

[156] Li J., Wang X., Zhang Y., Wei M., Qi J., Liu D., Wu R., Chen Q., Huang J. (2025). Ginsenoside Rg1 alleviates PCPA-induced insomnia by inhibiting NLRP3 inflammasome activation and pyroptosis through the Nrf2/HO-1 pathway in mice. Psychopharmacology.

[157] Wu Y., Zhang Z., Lian X. (2025). Ginsenoside Rc mitigates hippocampal neuronal damage and cognitive impairment in vascular dementia rats via the pY705-Stat3/Foxo3a and pS727-Stat3/GRIM-19 pathways. J. Ginseng Res..

[158] Yan X., Bai X., Sun G., Duan Z., Fu R., Zeng W., Zhu C., Fan D. (2024). Ginsenoside compound K alleviates brain aging by inhibiting ferroptosis through modulation of the ASK1-MKK7-JNK signaling pathway. Phytomedicine.

[159] Zhu Y., Li J., Dai L., Feng W. (2025). Ginsenoside Rh2 Alleviate Sepsis-related Encephalopathy via Up-regulating Nrf2/HO-1 Pathway and Apoptosis Inhibition. Cell Biochem. Biophys..

[160] Feng H., Xue M., Deng H., Cheng S., Hu Y., Zhou C. (2022). Ginsenoside and Its Therapeutic Potential for Cognitive Impairment. Biomolecules.

[161] Tavakkoli A., Iranshahi M., Hasheminezhad S.H., Hayes A.W., Karimi G. (2019). The neuroprotective activities of natural products through the Nrf2 upregulation. Phytother. Res..

[162] Divyajanani S., Harithpriya K., Ganesan K., Ramkumar K.M. (2023). Dietary polyphenols remodel DNA methylation patterns of NRF2 in chronic disease. Nutrients.

[163] Chen Y., Peng F., Xing Z., Chen J., Peng C., Li D. (2022). Beneficial effects of natural flavonoids on neuroinflammation. Front. Immunol..

[164] Khan H., Tundis R., Ullah H., Aschner M., Belwal T., Mirzaei H., Akkol E.K. (2020). Flavonoids targeting NRF2 in neurodegenerative disorders. Food Chem. Toxicol..

[165] Xu X., Li M., Chen W., Yu H., Yang Y., Hang L. (2016). Apigenin attenuates oxidative injury in ARPE-19 cells thorough activation of Nrf2 pathway. Oxid. Med. Cell. Longev..

[166] Moratilla-Rivera I., Sánchez M., Valdés-González J.A., Gómez-Serranillos M.P. (2023). Natural products as modulators of Nrf2 signaling pathway in neuroprotection. Int. J. Mol. Sci..

[167] Del Carmen García-Rodríguez M., Kacew S. (2025). Green tea catechins: Protectors or threats to DNA? A review of their antigenotoxic and genotoxic effects. Arch. Toxicol..

[168] Tang S., Zhang Y., Botchway B.O.A., Wang X., Huang M., Liu X. (2025). Epigallocatechin-3-Gallate Inhibits Oxidative Stress Through the Keap1/Nrf2 Signaling Pathway to Improve Alzheimer Disease. Mol. Neurobiol..

[169] Lai Z., Ke L., Zhao W. (2025). Naringenin as a neurotherapeutic agent in Alzheimer’s disease: Epigenetic signatures, gut microbiota alterations, and molecular neuroprotection. Front. Aging Neurosci..

[170] Mehranfard N., Ghasemi M., Rajabian A., Ansari L. (2023). Protective potential of naringenin and its nanoformulations in redox mechanisms of injury and disease. Heliyon.

[171] Solanki S., Vig H., Khatri N., Singh B.P., Khan M.S., Devgun M., Wal P., Wal A. (2025). Naringenin: A Promising Immunomodulator for Anti-inflammatory, Neuroprotective and Anti-cancer Applications. Anti-Inflamm. Anti-Allergy Agents Med. Chem..

[172] Kometsi L., Govender K., Mofo Mato E.P., Hurchund R., Owira P.M.O. (2020). By reducing oxidative stress, naringenin mitigates hyperglycaemia-induced upregulation of hepatic nuclear factor erythroid 2-related factor 2 protein. J. Pharm. Pharmacol..

[173] Rajappa R., Sireesh D., Salai M.B., Ramkumar K.M., Sarvajayakesavulu S., Madhunapantula S.V. (2018). Treatment with naringenin elevates the activity of transcription factor Nrf2 to protect pancreatic β-cells from streptozotocin-induced diabetes in vitro and in vivo. Front. Pharmacol..

[174] Wang K., Chen Z., Huang L., Meng B., Zhou X., Wen X., Ren D. (2017). Naringenin reduces oxidative stress and improves mitochondrial dysfunction via activation of the Nrf2/ARE signaling pathway in neurons. Int. J. Mol. Med..

[175] Davinelli S., Medoro A., Hu F.B., Scapagnini G. (2025). Dietary polyphenols as geroprotective compounds: From Blue Zones to hallmarks of ageing. Ageing Res. Rev..

[176] Sharifi-Rad J., Seidel V., Izabela M., Monserrat-Mequida M., Sureda A., Ormazabal V., Zuniga F.A., Mangalpady S.S., Pezzani R., Ydyrys A. (2023). Phenolic compounds as Nrf2 inhibitors: Potential applications in cancer therapy. Cell Commun. Signal..

[177] Farkhondeh T., Folgado S.L., Pourbagher-Shahri A.M., Ashrafizadeh M., Samarghandian S. (2020). The therapeutic effect of resveratrol: Focusing on the Nrf2 signaling pathway. Biomed. Pharmacother..

[178] Peng L., Hu X.Z., Liu Z.Q., Liu W.K., Huang Q., Wen Y. (2024). Therapeutic potential of resveratrol through ferroptosis modulation: Insights and future directions in disease therapeutics. Front. Pharmacol..

[179] Pourbagher-Shahri A.M., Farkhondeh T., Jafari-Nozad A.M., Darroudi M., Naseri K., Amirian M., Samarghandian S. (2024). Nrf2 Mediates Effect of Resveratrol in Ischemia-reperfusion Injury. Curr. Mol. Pharmacol..

[180] Scapagnini G., Vasto S., Abraham N.G., Caruso C., Zella D., Fabio G. (2011). Modulation of Nrf2/ARE pathway by food polyphenols: A nutritional neuroprotective strategy for cognitive and neurodegenerative disorders. Mol. Neurobiol..

[181] Shahcheraghi S.H., Salemi F., Peirovi N., Ayatollahi J., Alam W., Khan H., Saso L. (2021). Nrf2 regulation by curcumin: Molecular aspects for therapeutic prospects. Molecules.

[182] Cerullo M., Armeli F., Mengoni B., Menin M., Crudeli M.L., Businaro R. (2025). Curcumin Modulation of the Gut-Brain Axis for Neuroinflammation and Metabolic Disorders Prevention and Treatment. Nutrients.

[183] Kaur R., Sood A., Lang D.K., Arora R., Kumar N., Diwan V., Saini B. (2022). Natural Products as Sources of Multitarget Compounds: Advances in the Development of Ferulic Acid as Multitarget Therapeutic. Curr. Top. Med. Chem..

[184] Hassanein E.H.M., Althagafy H.S., Baraka M.A., Abd-Alhameed E.K., Ibrahim I.M., Abd El-Maksoud M.S., Mohamed N.M., Ross S.A. (2024). The promising antioxidant effects of lignans: Nrf2 activation comes into view. Naunyn Schmiedebergs Arch. Pharmacol..

[185] Siraj M.A., Islam M.A., Al Fahad M.A., Kheya H.R., Xiao J., Simal-Gandara J. (2021). Cancer chemopreventive role of dietary terpenoids by modulating Keap1-Nrf2-ARE signaling system—A comprehensive update. Appl. Sci..

[186] Kensler T.W., Egner P.A., Agyeman A.S., Visvanathan K., Groopman J.D., Chen J.-G., Chen T.-Y., Fahey J.W., Talalay P. (2012). Keap1–Nrf2 signaling: A target for cancer prevention by sulforaphane. Natural Products in Cancer Prevention and Therapy.

[187] Alves I., Araújo E.M.Q., Dalgaard L.T., Singh S., Børsheim E., Carvalho E. (2025). Protective Effects of Sulforaphane Preventing Inflammation and Oxidative Stress to Enhance Metabolic Health: A Narrative Review. Nutrients.

[188] Bessetti R.N., Litwa K.A. (2025). Broccoli for the brain: A review of the neuroprotective mechanisms of sulforaphane. Front. Cell. Neurosci..

[189] Habtemariam S. (2024). Anti-Inflammatory Therapeutic Mechanisms of Isothiocyanates: Insights from Sulforaphane. Biomedicines.

[190] Shah A., Varma M., Bhandari R. (2024). Exploring sulforaphane as neurotherapeutic: Targeting Nrf2-Keap & Nf-Kb pathway crosstalk in ASD. Metab. Brain Dis..

[191] Shannar A., Chou P.J., Peter R., Dave P.D., Patel K., Pan Y., Xu J., Sarwar M.S., Kong A.N. (2025). Pharmacodynamics (PD), Pharmacokinetics (PK) and PK-PD Modeling of NRF2 Activating Dietary Phytochemicals in Cancer Prevention and in Health. Curr. Pharmacol. Rep..

[192] Akaras N., Şimşek H., İleritürk M., Küçükler S., Gür C., Kandemir F.M. (2025). Carvacrol mitigates Mercury chloride induced neurotoxicity by regulation of NRF-2/HO-1/NF-κB expression. J. Trace Elem. Med. Biol..

[193] Shah S., Pushpa Tryphena K., Singh G., Kulkarni A., Pinjala P., Kumar Khatri D. (2024). Neuroprotective role of Carvacrol via Nrf2/HO-1/NLRP3 axis in Rotenone-induced PD mice model. Brain Res..

[194] Zhou H.L., Wang B.B., Fan X.L., Zhang X.M., Song Y. (2025). Carvacrol acetate activated Nrf2 modulates mitophagy for the treatment of neurocyte oxidative stress induced by chlorpyrifos. Ecotoxicol. Environ. Saf..

[195] Kathem S.H., Nasrawi Y.S., Mutlag S.H., Nauli S.M. (2024). Limonene Exerts Anti-Inflammatory Effect on LPS-Induced Jejunal Injury in Mice by Inhibiting NF-κB/AP-1 Pathway. Biomolecules.

[196] Kumar K.J.S., Vani M.G., Wang S.Y. (2022). Limonene protects human skin keratinocytes against UVB-induced photodamage and photoaging by activating the Nrf2-dependent antioxidant defense system. Environ. Toxicol..

[197] Jia L., Gong Y., Jiang X., Fan X., Ji Z., Ma T., Li R., Liu F. (2024). Ginkgolide C inhibits ROS-mediated activation of NLRP3 inflammasome in chondrocytes to ameliorate osteoarthritis. J. Ethnopharmacol..

[198] She W., Ma W., Zhang T., Wu X., Li J., Li X. (2025). Ginkgolide B inhibits ferroptosis in PC12 cells and ameliorates the oxidative stress in spinal cord injury through activating Nrf2 signaling pathway. J. Pharmacol. Sci..

[199] Verma H., Yadav A., Gangwar P., Kaur S., Kumar P., Dhiman M., Mantha A.K. (2025). A Cross-sectional In Vitro Study on the Synergistic Neuroprotective Effects of Phytochemicals Ferulic Acid and Ginkgolide B against Amyloid Beta-induced Oxidative Stress and Modulation of Multifunctional Enzyme APE1/Ref-1 in Human Neuroblastoma SH-SY5Y Cells. Cell Biochem. Biophys..

[200] Gjorgieva Ackova D., Maksimova V., Smilkov K., Buttari B., Arese M., Saso L. (2023). Alkaloids as Natural NRF2 Inhibitors: Chemoprevention and Cytotoxic Action in Cancer. Pharmaceuticals.

[201] Ashrafizadeh M., Fekri H.S., Ahmadi Z., Farkhondeh T., Samarghandian S. (2020). Therapeutic and biological activities of berberine: The involvement of Nrf2 signaling pathway. J. Cell. Biochem..

[202] Jing W., Safarpour Y., Zhang T., Guo P., Chen G., Wu X., Fu Q., Wang Y. (2018). Berberine upregulates P-glycoprotein in human Caco-2 cells and in an experimental model of colitis in the rat via activation of Nrf2-dependent mechanisms. J. Pharmacol. Exp. Ther..

[203] Rehman M.U., Rashid S., Arafah A., Qamar W., Al-Saffar R.M., Ahmad A., Almatroudi N.M., Alqahtani S.M.A., Rashid S.M., Ahmad S.B. (2020). Anti-cancer activity of piperine against colon carcinogenesis via modulation of NF-κB/Nrf-2/Keap1/HO-1 signalling pathways. Res. Sq..

[204] Li J., Ge H., Xu Y., Xie J., Yan F., Chen W. (2022). Geniposide Alleviates Oxidative Damage in Hepatocytes through Regulating miR-27b-3p/Nrf2 Axis. J. Agric. Food Chem..

[205] Wang Y.N., Li X.J., Wang W.F., Zou L., Miao H., Zhao Y.Y. (2024). Geniposidic Acid Attenuates Chronic Tubulointerstitial Nephropathy Through Regulation of the NF-ƙB/Nrf2 Pathway Via Aryl Hydrocarbon Receptor Signaling. Phytother. Res..

[206] Xiao X., Sun S., Li Y., Cen X., Wu S., Lu A., Cai J., Zhao J., Li S. (2022). Geniposide attenuates early brain injury by inhibiting oxidative stress and neurocyte apoptosis after subarachnoid hemorrhage in rats. Mol. Biol. Rep..

[207] Xiao Y., Zhang S., Ye Y., Chen J., Xu Y. (2023). Geniposide suppressed OX-LDL-induced osteoblast apoptosis by regulating the NRF2/NF-κB signaling pathway. J. Orthop. Surg. Res..

[208] Zhou Q., Chen B., Xu Y., Wang Y., He Z., Cai X., Qin Y., Ye J., Yang Y., Shen J. (2024). Geniposide protects against neurotoxicity in mouse models of rotenone-induced Parkinson’s disease involving the mTOR and Nrf2 pathways. J. Ethnopharmacol..

[209] Zhuge X., Jin X., Ji T., Li R., Xue L., Yu W., Quan Z., Tong H., Xu F. (2023). Geniposide ameliorates dextran sulfate sodium-induced ulcerative colitis via KEAP1-Nrf2 signaling pathway. J. Ethnopharmacol..

[210] Su L., Cao P., Wang H. (2020). Tetrandrine mediates renal function and redox homeostasis in a streptozotocin-induced diabetic nephropathy rat model through Nrf2/HO-1 reactivation. Ann. Transl. Med..

[211] Krajka-Kuźniak V., Baer-Dubowska W. (2021). Modulation of Nrf2 and NF-κB signaling pathways by naturally occurring compounds in relation to cancer prevention and therapy. Are combinations better than single compounds?. Int. J. Mol. Sci..

[212] Voulgaropoulou S.D., van Amelsvoort T.A.M.J., Prickaerts J., Vingerhoets C. (2019). The effect of curcumin on cognition in Alzheimer’s disease and healthy aging: A systematic review of pre-clinical and clinical studies. Brain Res..

[213] Godos J., Carota G., Caruso G., Micek A., Frias-Toral E., Giampieri F., Brito-Ballester J., Rodríguez Velasco C.L., Quiles J.L., Battino M. (2025). Molecular mechanisms underlying the neuroprotective effects of polyphenols: Implications for cognitive function. EXCLI J..

[214] Kassar O., ME M.M., Farag N., Selim A., Kewiaa Y., Yousef O., Hassan O. (2025). Efficacy and safety of sulforaphane in schizophrenia: A systematic review and meta-analysis of randomized controlled trials. BMC Psychiatry.

[215] Wang R., Ren Z., Li Y. (2025). The effect of sulforaphane on autism spectrum disorder: Systematic review and meta-analysis. EXCLI J..

[216] Cherian I.V., Vijukumar A., Islam M.M., Vikal A. (2025). Assessing the therapeutic potential of quercetin, a widely spread flavonoid, in the prevention and management of chronic and degenerative diseases through a modern Chinese medicine perspective. Pharmacol. Res. Mod. Chin. Med..

[217] Jin X., Liu M.Y., Zhang D.F., Zhong X., Du K., Qian P., Yao W.F., Gao H., Wei M.J. (2019). Baicalin mitigates cognitive impairment and protects neurons from microglia-mediated neuroinflammation via suppressing NLRP3 inflammasomes and TLR4/NF-κB signaling pathway. CNS Neurosci. Ther..

[218] Si L., An Y., Zhou J., Lai Y. (2025). Neuroprotective effects of baicalin and baicalein on the central nervous system and the underlying mechanisms. Heliyon.

[219] Zhai Z., Huang Y., Zhang Y., Zhao L., Li W. (2022). Clinical research progress of small molecule compounds targeting Nrf2 for treating inflammation-related diseases. Antioxidants.

[220] Gazaryan I., Thomas B. (2016). The status of Nrf2-based therapeutics: Current perspectives and future prospects. Neural Regen. Res..

[221] de Zeeuw D., Akizawa T., Audhya P., Bakris G.L., Chin M., Christ-Schmidt H., Goldsberry A., Houser M., Krauth M., Lambers Heerspink H.J. (2013). Bardoxolone methyl in type 2 diabetes and stage 4 chronic kidney disease. N. Engl. J. Med..

[222] Gold R., Kappos L., Arnold D.L., Bar-Or A., Giovannoni G., Selmaj K., Tornatore C., Sweetser M.T., Yang M., Sheikh S.I. (2012). Placebo-controlled phase 3 study of oral BG-12 for relapsing multiple sclerosis. N. Engl. J. Med..

[223] Linker R.A., Lee D.H., Ryan S., van Dam A.M., Conrad R., Bista P., Zeng W., Hronowsky X., Buko A., Chollate S. (2011). Fumaric acid esters exert neuroprotective effects in neuroinflammation via activation of the Nrf2 antioxidant pathway. Brain.

[224] Lynch D.R., Chin M.P., Delatycki M.B., Subramony S.H., Corti M., Hoyle J.C., Boesch S., Nachbauer W., Mariotti C., Mathews K.D. (2021). Safety and Efficacy of Omaveloxolone in Friedreich Ataxia (MOXIe Study). Ann. Neurol..

[225] Anand P., Kunnumakkara A.B., Newman R.A., Aggarwal B.B. (2007). Bioavailability of curcumin: Problems and promises. Mol. Pharm..

[226] Egner P.A., Chen J.G., Wang J.B., Wu Y., Sun Y., Lu J.H., Zhu J., Zhang Y.H., Chen Y.S., Friesen M.D. (2011). Bioavailability of Sulforaphane from two broccoli sprout beverages: Results of a short-term, cross-over clinical trial in Qidong, China. Cancer Prev. Res..

[227] Walle T. (2011). Bioavailability of resveratrol. Ann. N. Y. Acad. Sci..

[228] Sezgin-Bayindir Z., Losada-Barreiro S., Bravo-Díaz C., Sova M., Kristl J., Saso L. (2021). Nanotechnology-based drug delivery to improve the therapeutic benefits of NRF2 modulators in cancer therapy. Antioxidants.

[229] Rudrapal M., Mishra A.K., Rani L., Sarwa K.K., Zothantluanga J.H., Khan J., Kamal M., Palai S., Bendale A.R., Talele S.G. (2022). Nanodelivery of dietary polyphenols for therapeutic applications. Molecules.

[230] Naqvi S., Khanadeev V.A., Khlebtsov B.N., Khlebtsov N.G., Deore M.S., Packirisamy G. (2022). Albumin-based nanocarriers for the simultaneous delivery of antioxidant gene and phytochemical to combat oxidative stress. Front. Cell Dev. Biol..

[231] Jain V., Haider N., Jain K. (2018). 3D printing in personalized drug delivery. Curr. Pharm. Des..

[232] Jin X., Chen L., Yang Y., Tan R., Jiang C. (2025). Adverse effects of Nrf2 in different organs and the related diseases. Antioxid. Redox Signal..

[233] Robertson H., Dinkova-Kostova A.T., Hayes J.D. (2020). NRF2 and the ambiguous consequences of its activation during initiation and the subsequent stages of tumourigenesis. Cancers.

[234] Zimta A.-A., Cenariu D., Irimie A., Magdo L., Nabavi S.M., Atanasov A.G., Berindan-Neagoe I. (2019). The role of Nrf2 activity in cancer development and progression. Cancers.

[235] Cho H.-Y., Marzec J., Kleeberger S.R. (2015). Functional polymorphisms in Nrf2: Implications for human disease. Free Radic. Biol. Med..

[236] Ishikawa T. (2014). Genetic polymorphism in the NRF2 gene as a prognosis marker for cancer chemotherapy. Front. Genet..

[237] Godman B., Finlayson A.E., Cheema P.K., Zebedin-Brandl E., Gutiérrez-Ibarluzea I., Jones J., Malmström R.E., Asola E., Baumgärtel C., Bennie M. (2013). Personalizing health care: Feasibility and future implications. BMC Med..

[238] Jamalinia M., Weiskirchen R. (2025). Advances in personalized medicine: Translating genomic insights into targeted therapies for cancer treatment. Ann. Transl. Med..

